# First Transcriptome of the Testis-Vas Deferens-Male Accessory Gland and Proteome of the Spermatophore from *Dermacentor variabilis* (Acari: Ixodidae)

**DOI:** 10.1371/journal.pone.0024711

**Published:** 2011-09-16

**Authors:** Daniel E. Sonenshine, Brooke W. Bissinger, Noble Egekwu, Kevin V. Donohue, Sayed M. Khalil, R. Michael Roe

**Affiliations:** 1 Department of Biological Sciences, Old Dominion University, Norfolk, Virginia, United States of America; 2 Department of Entomology, North Carolina State University, Raleigh, North Carolina, United States of America; University of Minnesota, United States of America

## Abstract

Ticks are important vectors of numerous human diseases and animal diseases. Feeding stimulates spermatogenesis, mating and insemination of male factors that trigger female reproduction. The physiology of male reproduction and its regulation of female development are essentially a black box. Several transcriptomes have catalogued expression of tick genes in the salivary glands, synganglion and midgut but no comprehensive investigation has addressed male reproduction and mating. Consequently, a new global approach using transcriptomics, proteomics, and quantitative gene expression is needed to understand male reproduction and stimulation of female reproduction.

This first transcriptome to the reproductive biology of fed male ticks, *Dermacentor variabilis*, was obtained by 454 pyrosequencing (563,093 reads, 12,804 contigs). Gene Ontology (Biological Processes level III) recognized 3,866 transcripts in 73 different categories; spermiogenesis; spermatogenesis; peptidases, lipases and hydrolases; oxidative and environmental stress; immune defense; and protein binding. Reproduction-associated genes included serine/threonine kinase, metalloendoproteinases, ferritins, serine proteases, trypsin, cysteine proteases, serpins, a cystatin, GPCR and others. qRT-PCR showed significant upregulation from unfed versus fed adult male reproductive organs of zinc metalloprotease, astacin metalloprotease and serine protease, enzymes important in spermiogenesis and mating activity in insects, as well as a GPCR with the greatest similarity to a SIFamide receptor known to be important in regulating courtship behavior in *Drosophila*. Proteomics on these organs and the spermatophore by tryptic digestion/Liquid chromatography/Mass spectrometry/Mass spectrometry (LC/MS/MS) demonstrated expression of many of the same messages found by 454 sequencing, supporting their identification, and revealed differences in protein distribution in the reproductive system versus the spermatophore. We found Efα but no EF β in the transcriptome and neither of these proteins in the spermatophore. Thus, the previously described model for male regulation of female reproduction may not apply to other ticks. A new paradigm is needed to explain male stimulation of female tick reproduction.

## Introduction

Ticks are blood-feeding parasites that serve as vectors of the causative agents of many important diseases affecting humans and animals, e.g., Lyme disease, Rocky Mountain spotted fever, tick-borne encephalitis, anaplasmosis, babesiosis and many others [1]. In the hard ticks (Ixodidae), blood-feeding stimulates oogenesis and spermatogenesis. Blood-feeding also stimulates females to secrete sex pheromones that attract sexually active males for mating and insemination. During coitus, males rapidly assemble their spermatophores which fill with spermatids and seminal fluid [2]. Little is known about the composition of the spermatophores or how they are assembled. In females, mating and copulation induces the transition from slow to rapid feeding, followed by the cascade of reproductive events that eventually results in egg production. The identification of the male pheromone that triggers these profound physiological changes in the female of *Amblyomma hebraeum* was voraxin, consisting of two engorgement factor proteins [3]; however, to date, this function has not been confirmed in other tick species.

In insects, a remarkable array of proteins and peptides has been reported to occur in the seminal fluid of mating males [4]–[7]. Several of these proteins are known to regulate important female reproductive functions, e.g., by inducing or accelerating oocyte development, vitellogenesis, ovulation, oviposition and even reducing female sexual receptivity [4], [8]. Among the most significant is an accessory gland peptide (Acp), Acp70, also known as the “sex peptide” (SP) because of its role in stimulating both short-term and long-term post-mating responses; sperm is believed to be the carrier for SP. Studies show that SP binds to sperm [9]. Post-mating modification in the female body modulated largely by trypsin [10] is believed to release the mature peptide. The precise mode of action in the female is not fully resolved although critical new evidence suggests that it activates a G-protein coupled receptor in the brain [11]. Another important peptide in *Drosophila melanogaster* is Acp26Aa, synthesized in the male as a pro-hormone which is processed to the mature form during mating. It stimulates oviposition following its transfer to the female; however, its exact mode of action is unknown. This protein enters the hemolymph from the female genital tract, leaving open the possibility that, following further processing, Acp26Aa may act directly on the oviducts to induce peristalsis [12].

Another protein, a predicted astacin-like metalloprotease that occurs in the seminal fluid, is necessary to process Acp26Aa and a sperm storage protein, Acp36DE. Moreover, processing by the metalloprotease occurs in the body of the female [13]. In *Melanoplus sanguinipes* and *Locustra migratoria*, oviposition-stimulating proteins induce egg laying when injected directly into the hemolymph [4]. In *Rhodnius*, the male accessory gland (MAG) secretions are believed to contain a myotropin-inducing peptide that acts on neurosecretory cells in the brain to secrete myotropin; the latter triggers ovulation and oviposition in mated females (reviewed by [14]).

Additional factors have been found in the male seminal fluid. For example, the male sex peptide stimulates increased female food uptake [15]. In crickets, prostaglandin E_2_ synthetase from the male, introduced during copulation, stimulates ovarian development and oviposition [16]. It is of interest that PGE2 is present in tick saliva and is known to have important effects in modulating host responses during feeding [17]. Tick saliva is also secreted during copulation and lubricates the spermatophore for insemination. The complexity of the Acps is substantial, presenting a daunting challenge for understanding the variety of effects induced by the seminal fluid in insects. Much less is known about the receptors in mated females or how they respond to sex proteins from the copulating male.

Compared to insects, little is known about the molecular biology of spermatogenesis in ticks and the male regulation of female reproduction. The genes involved in the transformation of the spermatogonia into fully capacitated, elongated spermatids [18], in the formation of the spermatophore and its seminal contents and especially those that stimulate the females to engorge and reproduce are unknown. A peptidic male engorgement factor (EF) inseminated into mated females was reported to stimulate full engorgement in the bont tick, *Amblyomma hebraeum*. This pheromone was shown to consist of two peptides, Efα and Efβ, produced in the testis/vas deferens (TVD) but not in the male accessory gland (MAG) in that species [3]. However [19], found only EFα in *Dermacentor variabilis* and showed that knockdown of this gene by injection of the complementary dsRNA into unfed males (i.e., before the normal expression of EFα in fed males) did not disrupt female engorgement to repletion when these males were allowed to freely mate. In view of the paucity of information about the biology of male tick reproduction, these studies were undertaken to address the factors that regulate spermatogenesis and, possibly, affect post-coital female reproduction.

Here we describe the transcripts from the testes-vas deferens-male accessory gland of fed, *D. variabilis* males and the spermatophore proteome to advance our understanding of the male reproductive system and its role in the regulation of female reproduction.

## Results and Discussion

### 3.1. Assembly, annotation and functional analysis

Pyrosequencing resulted in 563,093 raw reads which were assembled into 12, 804 contigs. Read length of transcripts ranged from 74 to 6003 bp with an average contig length of 299.8 bp. Most (72.1%) were less than 250 bp (using the older sequencing reagents as noted earlier). A total of 3,951 contigs were found with e-values≤e-10 (30.9%) when compared to the GenBank NR database using BLASTx.

**Figure 1:**
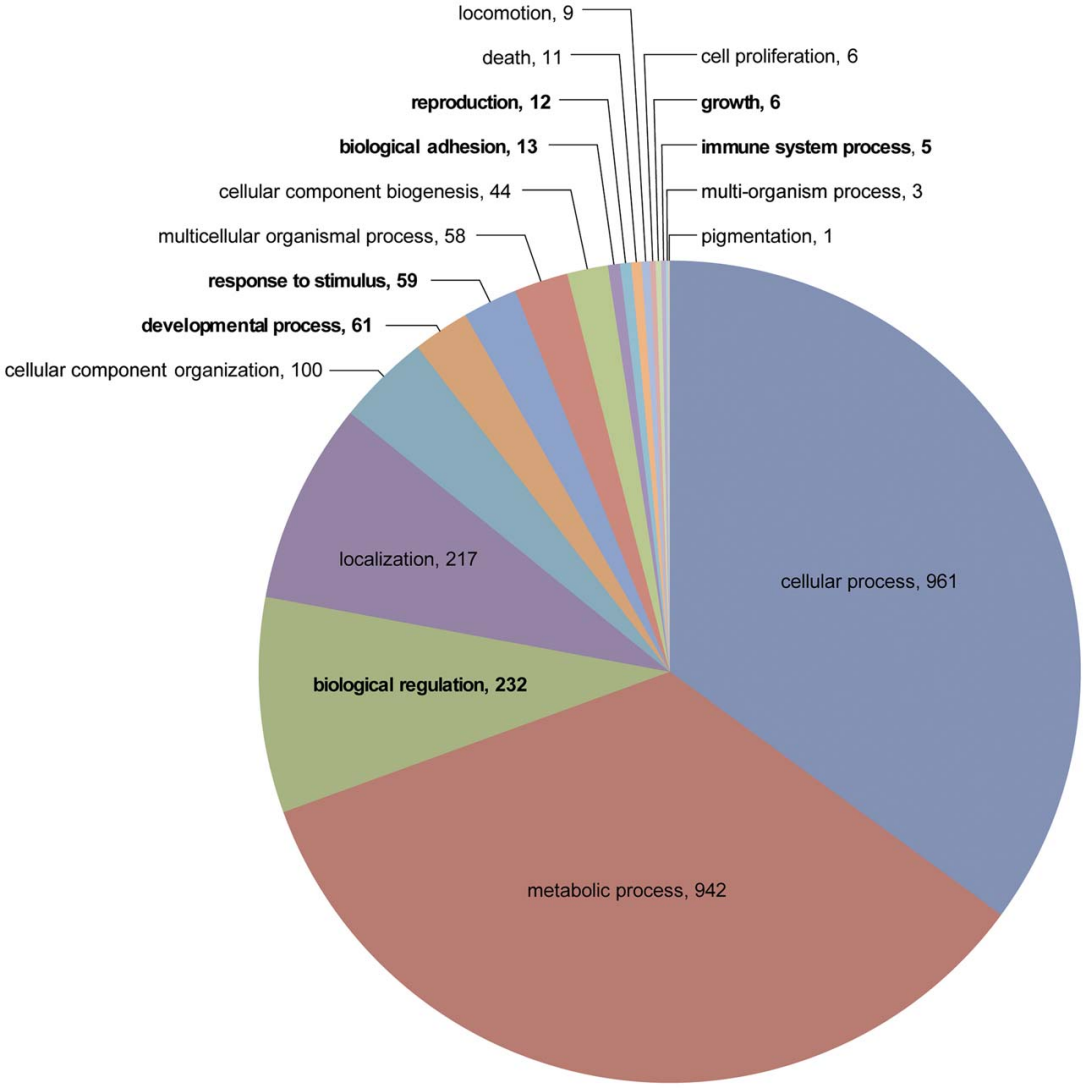
Analysis of *D. variabilis* male transcriptome based on GO level II biological processes. Level II functional assignments comprised 2740 contigs in 18 separate categories.

**Figure 2:**
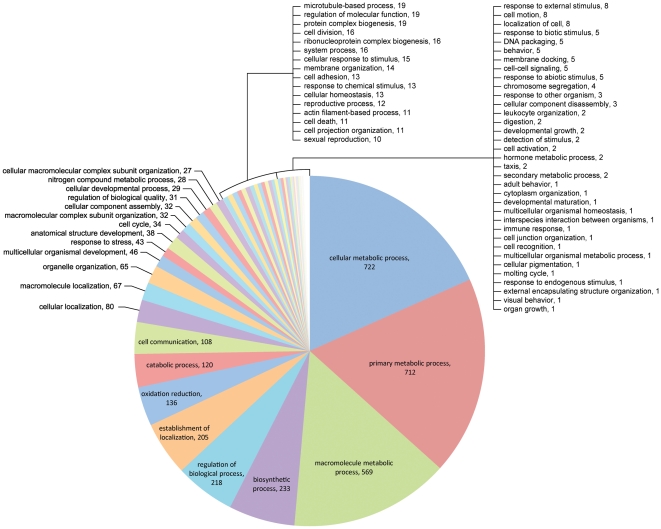
Analysis of *D. variabilis* male transcriptome based on GO level III biological processes. Level III functional assignments comprised 3,898 contigs in 73 separate categories. The terms in bold are believed to be those most relevant to spermatogenesis and male stimulation of female reproductive activity.

Of the contigs with BLASTx e-values≤e-10 matches, a total of 3,345 contigs in the library had Gene Ontology (GO) assignments categorized by their biological processes (BP) function (Figures 1, 2). Fig. 1 shows the 2,740 contigs assigned to the 18 BP level II categories, including broad class biological properties such as reproduction, biological adhesion or developmental processes. Figure 2 shows the 3,898 GO contigs assigned (some of the same contigs were assigned to more than 1 category) in 73 level III categories, including additional broad class biological properties as reproductive activity, oxidation/reduction, developmental growth and others as described below. The GO categories believed to be of greatest importance for tick spermatogenesis and possible stimulation of female engorgement included cell adhesion (2.8%), cell communication (0.3%), cellular response to stimulus (0.4%), immune response (0.03%), oxidation reduction (3.5%), reproductive processes (0.3%), response to abiotic stimulus (0.1%), response to chemical stimulus (0.3%) and sexual reproduction (0.3%). Most (69.5%) were associated with basic cellular and metabolic functions. At BP level II, only 12 contigs were assigned to reproductive functions, 13 to protein-cell adhesion and only 5 to immune functions (slightly more than1%). However, when expanded to BP level III, a total of 3,866 contigs could be assigned to 73 categories in this list. In addition, 3,926 contigs did not match any known sequence (data not shown). Of special interest is the much larger number of contigs assigned to reproduction/reproductive processes (22), biological/cellular adhesion (12), response to stress (43), oxidation/reduction (136) and response to stimuli (18). Proteases involved in digestion, membrane regulatory functions and protease inhibitors are included within the catabolic processes and other categories; these will be described separately later. Proteins with these types of functions have been recognized as components of the seminal fluids of insects [8]–[20], and several have been implicated in stimulating females to commence reproductive activity.

### 3.2. The most abundant contigs identified from the male transcriptome

Table S1 lists the 50 most abundant contigs (each have ≥573 reads) in the cDNA library of the MAG/TVD with e-values≤e-10; exceptions are included for highly abundant contigs with higher e-values where matches to the same proteins identified by LC MS/MS were found in the male accessory gland/testis vas deferens (MAG/TVD) and/or the spermatophore. Contigs (3,926) with no sequence similarity to genes in GenBank were excluded. The occurrence of transcripts with homologies to the same gene is likely the result of incomplete sequences that did not overlap and consequently were not assembled into a contiguous sequence. No significant correlation was found between transcript length (bp) and sequencing frequency (number of reads) by regression analysis using the SAS procedure PROC REG (SAS® version 9.1, SAS Institute, Cary, NC) (*t* = −0.23, *P* = 0.82).

Of special interest with regard to spermatogenesis and copulation are the contigs for serine/threonine kinase, metalloproteases, ferritin, heat shock protein and serine protease (see references following). Contig 12738, a serine/threonine kinase, was sequenced 3410 times. Three other contigs matching this function, 12696, 12749 and 11854, were sequenced 2135, 748 and 590 times, respectively. qRT-PCR also showed high expression of serine/threonine protein kinase (Tables 1 and 2, contig 11582). Serine/threonine protein kinase was also identified by LC MS/MS in the MAG/TVD. In humans, serine/threonine protein phosphorylation is believed to have a physiological role in sperm capacitation [21]. In vertebrates, serine/threonine kinases are believed to function in the reorganization of sperm chromatin during spermiogenesis [22]. Its abundance in the MAG/TVD suggests that it may have an important role in male reproductive activity. A serine/threonine kinase protein was also identified by LC MS/MS in the MAG/TVD but not in the spermatophore (Table S2).

**Table 1:** Expression of selected genes of interest from the male MAG/TVD transcriptome in response to feeding/courtship^1^ as determined by quantitative real-time PCR.

Category	Gene	Contig	Fold^2^	*P*-value	Significance
Reproduction	Astacin metalloprotease	03261	7.06:1	1.91	*P* = 0.20
“	Serine/threonine kinase	11582	1.61:1	0.49	*P* = 0.48
“	Zinc metalloprotease	00843	5.01:1	6.23	*P* = 0.016
Proteases	Cathepsin B	00689	1.03:1	0.14	*P* = 0.90
“	Cysteine protease^3^	05033	0.21:1	3.08	*P* = 0.05
“	Serine protease	12380	22.0:1	12.39	*P* = 0.003
“	Trypsin	03705	13.5:1	5.45	*P* = 0.003
Protease inhibitor	Serine protease inhibitor	11029	0.10:1	7.31	*P* = 0.003
Oxidase stress	Thioredoxin	09407	1.13:1	0.49	*P* = 0.63
Structure/adhesion	Calreticulin	10500	0.39:1	2.29E-7	*P* = 0.001
“	Keratin	01860	0.89:1	5.14E-12	*P* = 0.001
“	Laminin	12680	0.45:1	0.0005	*P* = 0.001
“	Tetraspanin	10467	2.93:1	0.002	*P* = 0.001
Lipases	Phospholipase C	06555	1.20:1	0.64	*P* = 0.58
Neuropep. Receptor^4^	GPCR	08424	2.97:1	3.54	*P* = 0.002

1 Transcriptome of extract made from fed *Dermacentor variabilis* male accessory glands, testis and vas deferens. Males were exposed to females (defined as courtship) but were not allowed to copulate. Expression compares fed versus unfed males for the MAG/TVD.

2 Change in fed male versus unfed male MAG/TVD.

3 Equals Cathepsin L.

4 Neuropeptide Receptor.

[The individual assays for these qPCR results can be supplied by authors upon request].

**Table 2:** List of primers used for qRT-PCR.

Gene Name	Contig No	Forward/Reversed	Sequence
Zinc Metalloprotease(ZM)	00843	(ZM) Forward	5′-CGA GAG CGG CAC TGC AA-3′
“		(ZM) Reversed	5′-GTG CTG CCG TCG ACC AA-3′
Serine/threonine kinase (SK)	11582	(SK) Forward	5′-CAA TGG GAG GCA TTC AAA GC - 3′
“		(SK) Reversed	5′-GCT CCT CCA TGT TGG ATT GGT - 3′
Astacin-like metalloprotease (AM)	03261	(AM) Forward	5′-CGT TCG GAC AGG GAC CAA -3′
“		(AM) Reversed	5′-TTC TGG CTC CGC GTT CTC-3′
Cathepsin B (CB)	00689	(CB) Forward	5′-TCG CCT GCA CGA GGA AA -3′
“		(CB) Reversed	5′-CGG GCG TCG AAC GAT TC -3′
Cysteine Protease (CL)	05033	CL) Forward	5′-GGGCCAGCATTTCCTGAA-3′
“		(CL) Reversed	5′-GAGCAGTCGACCAAGTTTTGC-3′
Serine protease (SP)	12329	(SP) Forward	5′-GGA TTC AGG CGG ACC GTT A-3′
“		(SP) Reversed	5′-CCG ACT TGC AGC GTT CTT C-3′
Trypsin(T) Trypsin-like serine protease	03705	(T) Forward	5′-CAC CAC CGT GAC CTT TTG TG -3′
“		(T) Reversed	5′-TGC GGC GCA CTG CTT -3′
Serine Protease inhibitor (serpin)	11029	(Serpin) Forward	5′-TCA TCG CGT GCA ACA TAA AG-3′
“		(Serpin) Reversed	5′-GAG CAC AGA TAC TGC GAA AT-3′
Thioredoxin (TH)	09407	(TH) Forward	5′-CTC CAA GCC GGC TCC ATA-3′
“		(TH) Reversed	5′-TGA GCT CTT TGA ACT CGC CAT T-3′
Calrecticulin (CR)	10500	(CR) Forward_1_	5′- CCC TGG TCG GCT TAA TATCG-3′
“		(CR) Reversed_1_	5′-CCA TCGTTG AAC TCT TCT TTG AAG -3′
“		(CR) Forward_2_	5′-CAA CCTCGG AAA GTT CGT TCT C-3′
“		(CR) Forward_2_	5′-AGT CCCTTG CTT TTC TCC TCA TC -3′
Keratin (K)	01860	(K) Forward	5′-GCA GTACCT CTA CCA CTT CCT TGT C - 3′
“		(K) Reversed	5′- GCT GTT CAG GTT CAC TTC AGT CA - 3′
Tetraspanin (TS)	10467	(TS) Forward	5′-TGC GGC ATT GCC TTG ATA G-3′
“		(TS) Reversed	5′-TGT GGG CTT AGG GCT GTG A-3′
Laminin (LM)	12680	(LM) Forward	5′-CTC TCG CGT CAG CAT CCA-3′
“		(LM) Reversed	5′-CTC TCC CAT GCA ACA ACA AG-3′
Phospholipase A	06555	(PL) Forward_1_	5′-TCG GTC TGG CGT CTG GTA A-3′
“		(PL) Reversed_1_	5′-TCC CGT CGA CTT GTG GAA-3′
“		(PL) Forward_2_	5′-CGC CGT TCA CTT TCC TCT AAA-3′
“		(PL) Reversed_2_	5′-TGC ATT CAG CGC CTG AGA-3′
G-coupled protein receptor (GCPR)	08424	(GPCR) Forward	5′-GCG CAC GGT CAC CAA CTA T-3′
“		(GPCR) Reversed	5′-GAA CAC GAC GAC CAG GAT GTC-3′

Transcripts with homology to genes for transmembrane proteins also were abundant. Transmembrane proteins appear to be important in spermiogenesis and possibly other male reproductive functions [23], [24] although they are not exclusive to the reproductive system. Metalloendoproteases are associated with post-translational modification as well as protein turnover [25], [26]. Transcript 11512, with a functional assignment to neprilysin, a membrane metalloprotease reported to be important in sperm function and normal fertilization in *D. melanogaster* [27], was sequenced 1246 times. Other transmembrane proteins sequenced with high frequency include contig 11464, , also with homology to metalloendopeptidase; contig 12307, , a transmembrane protein reported to regulate sperm motility; and contig 11934, with homology to a gene for neprilysin-like metalloendopeptidase activity. Neprilysin sperm-associated metalloprotease protein also was identified in the fed male spermatophore but not in the MAG/TVD by LC MS/MS (Table S3, Reproduction/reproductive processes). Contig 12307 showed homology to transmembrane protein, a cell surface low density lipoprotein receptor believed to be important in sperm maturation and cell signaling (www.uniprot.org, Q14114). The large number of metalloproteinases and high frequency of expression suggests that these merit further study to determine their roles in male reproduction.

**Figure 3:**
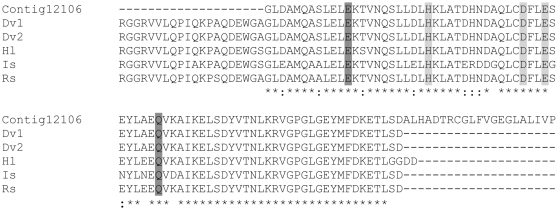
Multiple sequence alignments for putative ferritin from *D. variabilis* male transcriptome versus other species. Multiple sequence alignment (ClustalW) of the deduced amino acid sequence of a putative *D. variabilis* ferritin (Contig12106) identified from the 454 transcriptome to the male reproductive system and other putative tick ferritins. *Dermacentor variabilis* (Dv1, AAL75582; Dv2, AAQ54712), *Ixodes scapularis* (Is; AY277906), *Haemaphysalis longicornis* (Hl, AAQ54713), *Rhipicephalus sanguineus* (Rs; AY277907). Dark grey shading represents two of seven residues conserved among the ferroxidase di-iron center. Light grey shading indicates the three residues that characterize the iron ion channel. Asterisks denote identical residues, dots indicate conservative substitutions.

Two contigs, 12106 and 12619, had high homology (100%) to *D. variabilis* ferritin (AAL75582). Ferritins are important iron storage proteins and are expressed in highly metabolically active tick body organs and tissues [28]. Their abundance in the MAG and testis may be an important indicator of the high metabolic activity of these organs during reproduction [29]. According to Law [30], [31], iron is a vital nutrient in insects. Alignment of contig 12106 shows high sequence similarity to other tick ferritins (4.0e-38, 73% similarity, Figure 3) and is also similar to contig DvM 131 reported by [62] in the midgut of this same species (Figure 3). Ferritins may also function as antimicrobials [28], [31]. Ferritins additionally are important in blood-feeding and reproduction in ticks; knockdown (by RNAi) of two ferritin genes (*fer1* and *fer2*) resulted in decreased oviposition, egg hatching and post-bloodmeal weight in females of the sheep tick, *I. ricinus* [29]. Whether this would affect reproduction in male ticks is unknown.

**Figure 4:**
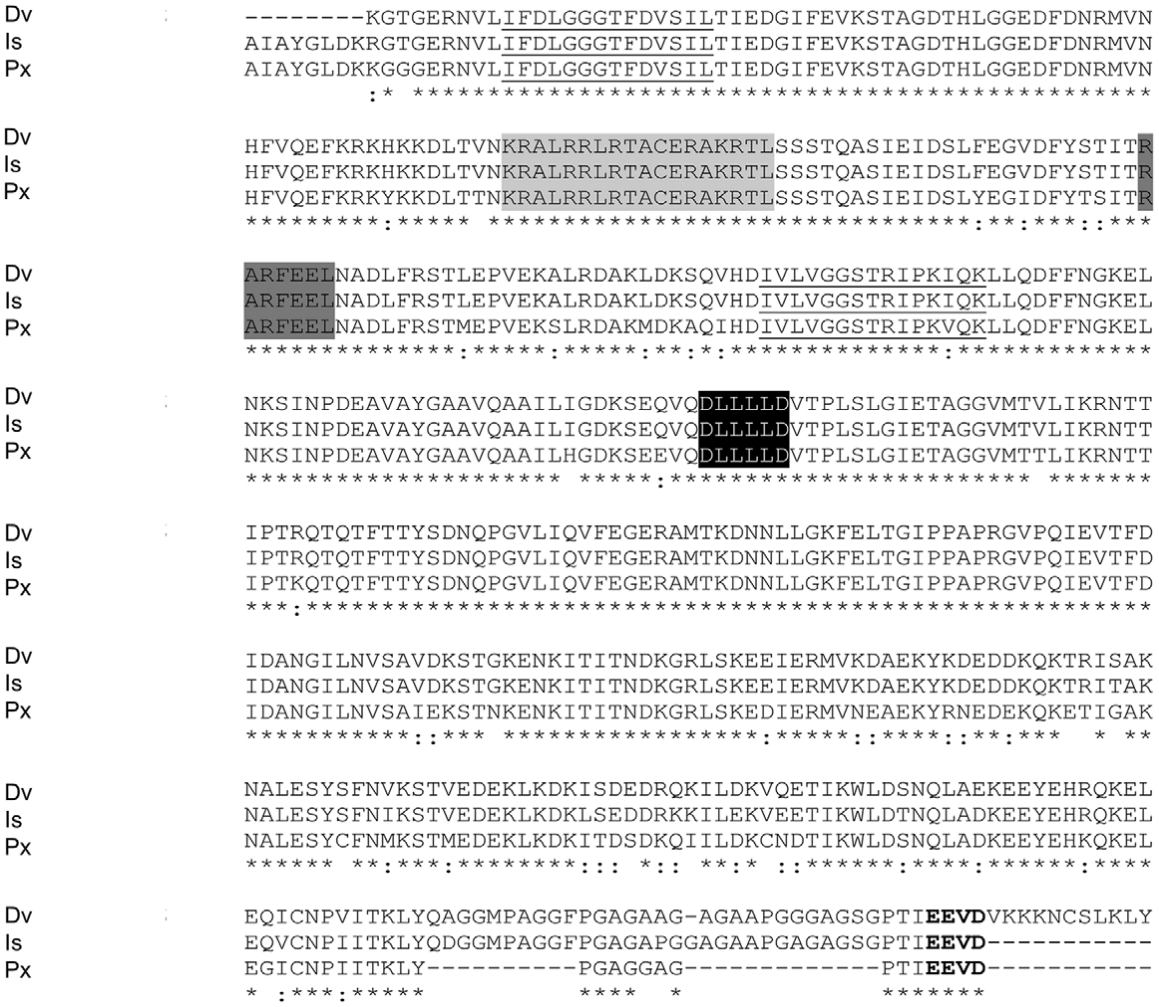
Multiple sequence alignments for heat shock protein from *D. variabilis* male transcriptome versus other species. Multiple sequence alignments (ClustalW) of the deduced amino acid sequence of a putative *D. variabilis* Hsp70 with other putative Hsps from the Ixodida are compared. *Dermacentor variabilis* (Dv; contig11387), *Ixodes scapularis* (Is; EEC03688), *Plutella xylostella* (Px; BAE48743). Underlined residues show two of the three signature sequences of Hsp70 [78]. Residues shaded in light grey indicate one of the two bipartite nuclear localization signals common in Hsp70 [78]. Dark grey signifies the non-organellar consensus motif [79]. The linker region between ATPase and peptide domains is shaded in black [80]. The conserved EEVD motif at the C-terminus is bolded [78]. Asterisks denote identical residues, two dots signify conservative substitutions.

Another highly expressed contig was heat shock protein (Hsp) (contig12677). Other contigs of this same protein but sequenced less frequently are noted in Table S10 with their annotation as Hsp supported by the highly conserved alignment with other species and the presence of highly conserved amino acids (Figure 4). Heat shock proteins were also identified in the protein extracts of the MAG/TVD and spermatophore by LC MS/MS (Table S2). Hsps are typically upregulated in response to stress but may also function in other roles, e.g., as molecular chaperones [32]. Mulenga et al. [33] reported Hsp expression in *A. americanum* in response to exposure to host animals as an example. Nothing is known of their role in male tick reproduction. In the present study, 39 putative Hsp contigs were sequenced from the male reproductive system transcriptome (although only 3 had very low e-values and were considered for inclusion in this report).

**Figure 5:**
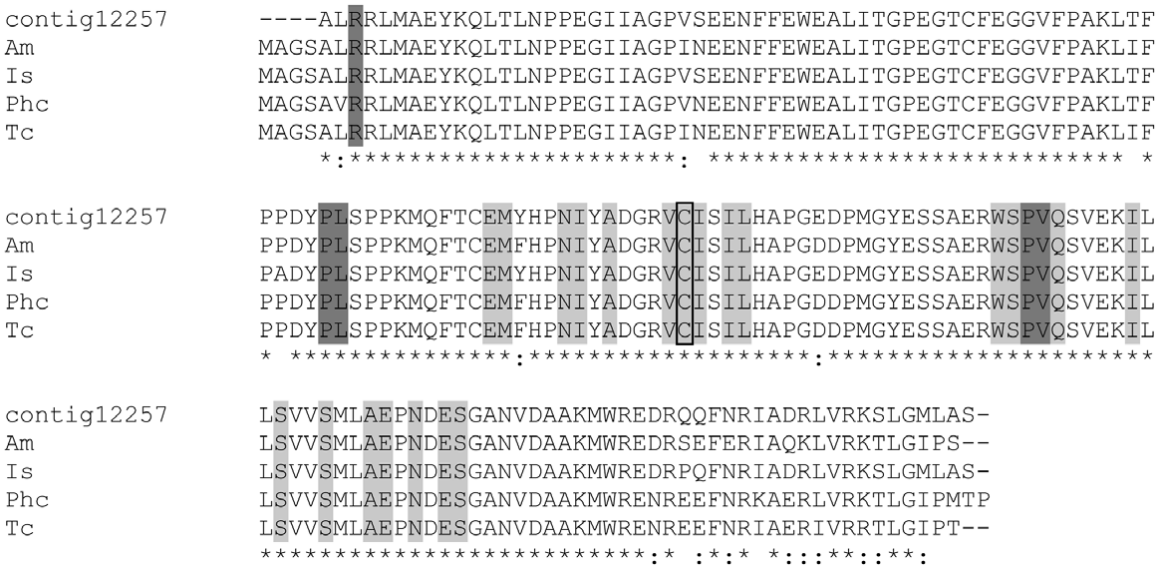
Multiple sequence alignments for ubiquitin conjugating enzyme from *D. variabilis* male transcriptome versus other species. Multiple sequence alignment (ClustalW) of the deduced amino acid sequence of a putative *D. variabilis* ubiquitin-conjugation enzyme (UBC) E2 catalytic domain (Contig12257) from the 454 transcriptome to the male reproductive system and published UBCs from the Arthropoda are compared. *Apis mellifera* (Am; XP_625157), *Ixodes scapularis* (Is; EEC09803), *Pediculus humanus corporis* (Phc; XP_002430814), *Tribolium castaneum* (Tc; XP_973689). Light grey shading indicates the 21 residues involved in the ubiquitin thioester intermediate interaction on the ubiquitin-conjugating enzymes catalytic domain (UBCc). The Cys in the active site of the UBCc conserved domain is boxed. Dark grey shading denotes the five E3 interaction residues on the UBCc. Asterisks denote identical residues, dots indicate conservative substitutions.

Ubiquitin was highly expressed in the male reproductive transcriptome. Five contigs with significant homology to ubiquitin conjugating enzyme (contigs 12709, 12640, 12257, 12428 and 11880) were sequenced multiple times. Its identification is supported by the alignment of similar sequences from *I. scapularis* and insects (Figure 5) as well as by the identification of ubiquitin in the protein extracts of the fed male reproductive tissues and spermatophore with a match to a similar protein in *Drosophila* (NP_476776, *D. melanogaster*) and multiple copies of ubiquitin ligase (e.g., GB: AAY66893, *I. scapularis*; GB: ACX54040, *R. sanguineus*). Ubiquitin mediates the degradation of regulatory proteins. It is important in regulating protein turnover, apoptosis, development, immune response and many other cellular functions [34].

Serine protease was abundantly expressed in the fed male reproductive system. Serine proteases are proteolytic enzymes which, like the metalloproteases in the male seminal fluid noted previously, may have important post-coital functions in the body of the female. The most noteworthy example is the reported increased expression of hemolymph trypsin (a serine protease) in *Drosophila sp* females following mating, which triggers the release of the mature sex peptide inseminated by the male into the body of the female [10]. In the present study, analysis by qRT-PCR showed that serine protease was strongly upregulated (22-fold) in the fed male versus the unfed male reproductive system, whereas serine protease inhibitor was strongly down regulated (Table 1). Serine protease and trypsin were identified by LC MS/MS in the MAG/TVD and spermatophore (Table S2). Thus, it is possible that these proteins were secreted into the semen. Most of the remaining contigs in the top 50 most highly expressed genes were putative housekeeping genes, e.g., cytochrome C oxidase and NADH dehydrogenase.

### 3.3. Proteins (peptides) in the spermatophore and MAG/TVD

**Figure 6:**
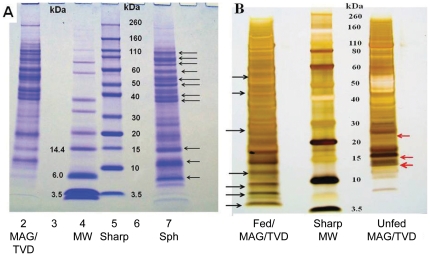
Protein gels showing contrasts between *D. variabilis* reproductive organs and spermatophore. **A**. Image of Coomassie-stained gel electrophoresed under reducing conditions showing the contrasts between proteins in the fed male accessory gland/testis vas deferens (MAG/TVD) and the spermatophore. Protein loading was 40 µg in lane 2 (MAG/TVD) versus 15 µg in lane 7 (spermatophore). Arrows indicated the most intense bands in the spermatophore. **B**. Image of a Silver –stained gel showing the contrasts between proteins from extracts of fed versus unfed male accessory gland/testis vas deferens. Protein loading was 15 µg in both lanes with extracts from fed MAG/TVD versus unfed MAG/TVD. Black arrows indicated the most intense bands in the extract of the fed male but absent (or weakly expressed) in the extract of the unfed male. Red arrows indicate most intense bands in the extract of the unfed male but absent in the fed male.

Figure 6A shows a representative Coomassie Blue-stained gel of the proteins from the male reproductive system and spermatophore. At least 21 bands are evident in the lane with the spermatophore, ranging from 5–110 kDa; 19 bands were evident in the MAG/TVD ranging from 6–100 kDa. Eleven bands in the spermatophore were strongly expressed, including bands at 6, 11, 14, 37, 39, 50, 55, 59, 67, 79 and 100 kDa (indicated by black arrows). The bands at 5 and 6 kDa appear to be unique to the spermatophore while the bands at 11and 37 kDa are much more strongly expressed than in the MAG/TVD. Proteins at approximately 10, 12–14 and 37 kDa excised from the MAG/TVD lane and 6 and 11–12 kDa from the spermatophore were submitted for protein identification by LC MS/MS. These regions were selected based on estimates in the literature for the molecular weights of male accessory gland proteins from *Drosophila* spp. and other insects [7], [35]. Figure 6 B shows a comparison of the protein bands present in the MAG/TVD from fed versus unfed males. Note the 4 bands ranging from approximately 5–12 kDa present in the fed male (black arrows) but absent in the unfed male and also the 3 bands ranging from approximately 13–25 kDa (red arrows) in the unfed male but apparently absent in the fed male. These differences may indicate that certain proteins are upregulated while others are downregulated in response to feeding.

Table S2 shows 56 of the more than 900 peptides in the spermatophore and/or MAG/TVD identified by tryptic digestion/LC-MS/MS and their corresponding matches to contigs in the transcriptome. Most (73%) of the 56 peptides had the same or very similar putative identifications as the contigs in the transcriptome. For simplicity, housekeeping proteins were excluded. Space does not allow for discussion of all of the proteins identified. The 56 selected peptides were categorized into 9 groups, presumptively involved in spermatogenesis, mating and copulation as follows:

**(1) Reproduction/reproductive proceses.** Among the most important of the 12 identified proteins in this group are the following: (a) Neprilysin, abundant in the *D. melanogaster* nervous system and testes [36]. Most neprilysins are membrane-bound enzymes that hydrolyze signaling peptides (among other functions); some are also soluble after proteolytic cleavage [37]; (b) ADAM (a-disintegrin-metalloprotease), a sperm surface enzyme important for normal fertility [27]. These proteins also act at the cell surface to trigger signaling activity, cell adhesion and other functions [38]; (c) angiotensin converting enzyme (ACE) ( = Dipeptidyl carboxypeptidase II), known to be important for fertilization of the eggs by the sperm [39]. ACE is expressed in the male accessory glands in *D. melanogaster* in the electron dense granules of the secondary cells but is lost from the glands during mating, consistent with transfer to the female via the seminal fluid [40]; (d) arylsulfatase, associated with acrosomal development in the spermatids [41]; and (e) cyclophilin A, a protein involved in blood ingestion, blood meal processing and subsequent oocyte development. It is believed to work by mediating the folding of essential proteins for oocyte development [42]. (2) *Peptidases*. Peptidases were reported to function in various aspects of male reproduction. Eleven peptidases were found including trypsin protease, carboxypeptidase, dipeptidyl carboxypeptidase, serine protease, leucine aminopeptidase and cathepsin L-like cysteine protease; qPCR showed significant up-regulation of serine protease and trypsin, significant down-regulation of cathepsin-L, but no evidence of change in the cathepsin B (cysteine protease B) (Table 1). (3) *Protease inhibitors*. Included are cystatin or cysteine protease inhibitor, aprotinin and trypsin inhibitor. (4) *Hydrolases* found included mannosyl-3-phosphoglycerate phosphatase, lactate dehydrogenase and lactamase domain protein. (5) *Lipases* found included three different phospholipases. (6) *Oxidative stress proteins* included quinone oxireductase, pyridine nucleotide disulphide oxidreductase, superoxide dismutase, glutathione S-transferase, thioredoxin and others. (7) *Environmental stress proteins* all of which were heat shock proteins (HSP 70, HSP 90 and others). (8) *Immune proteins* including lysozyme, macrophage migration inhibitory factor and ML-domain protein. (9) *Cell adhesion-related proteins* (including actin, alpha and beta-tubulin, cytokeratin, keratin and calreticulin and others). Unexpected was the discovery of the alpha and beta hemoglobin in both the MAG/TVD and the spermatophore. It is possible that their presence in the MAG/MTV resulted from host blood contamination during dissection or from hemolymph contamination. However, the presence of hemoglobin in the spermatophore suggests that the proteins are sequestered and secreted by the MAG/MTV either for a specific function or as a host contaminant incorporated during the formation of this structure. Fragments resulting from hemoglobin digestion have been shown to have antimicrobial activity in ticks [43], [44] and could have a similar role in the spermatophore and female genital tract which may explain their presence in the MAG and spermatophore.

We did not find any evidence of the peptides for the male engorgement factor (Efα and/or Efβ), the two proteins that were reported by Weiss and Kaufman [3] to comprise voraxin. Voraxin is the factor produced by the male that stimulates *Amblyomma hebraeum* females to engorge following copulation. We found the contig for Efα in the *D. variabilis* MAG/TVD transcriptome (see below section 3.4.1) but no evidence of Efβ. Numerous other proteins identified in these structures can be found by searching the attached file.

### 3.4. Supporting Tables and Figures

Numerous additional contigs with lower numbers of reads that are potentially important in male reproduction are discussed below. Supporting tables listing these contigs and figures illustrating alignments are provided as **Supporting Information (tables S1, S2, S3, S4, S5, S6, S7, S8, S9, S10, S11, and S12 and figures S1, S2, S3, S4, S5, S6, S7, and S8)**. Contigs discussed previously are excluded.

#### 3.4.1. Reproductive activity

Twelve contigs were categorized by the GO program as reproduction-related proteins or peptides. However, manual annotation of sequences in the transcriptome identified 16 contigs that could be assigned specifically to this category (Table S3). Of special interest are the following: (1) *26S protease sperm-associated protein*, a member of a family of putative ATPases associated with the 26S proteasome complex [45]; (2) *Prostaglandin F synthase*. Prostaglandins (PG) are known to have profound stimulatory effects on the physiological activity of different tissues throughout the animal kingdom [46]. Contig 07031 showed a match to a prostaglandin F synthase (e-value 5.0-18). However, alignment of the deduced amino acid sequence with similar sequences from *I. scapularis* and insects did not show the characteristic domains or other evidence of conserved amino acids characteristic of this protein (alignment not shown). In the tick *A. americanum*, high concentrations of PGE2 were found in salivary glands, tick saliva and hemolymph [47]. PGE2 is responsible for the secretion of tick salivary gland proteins via a phosphoinositide signaling pathway and mobilization of intracellular Ca^2+^ [48]. In crickets, this enzyme is found in the spermatophore but absent in virgin females. Following insemination, PG synthase activity was found in the mated females where it induced oviposition [16]. The possible presence of prostaglandin synthesis enzyme in the tick MAG/TVD or secretion of PG synthase and/or PGE2 in the saliva is a candidate for further study as a possible factor stimulating female reproductive activity. It should be noted that males secrete saliva to lubricate the female vulva during copulation and prior to inserting the spermatophore; (3) *9.8 and 10.4 kDa basic proteins*. Contigs 1950 and 10624 matched similar peptides identified in the male reproductive organs of *A. hebraeum* and were reported to be important in spermatogenesis in that species. Males in which these genes were knocked down by RNAi demonstrated abnormal spermiogenesis, and females mated to these males demonstrated reproductive failure [49]. A BLAST search showed that contig 1950 matches a 9.8 kDa protein in *A. hebraeum* (e-value 4.0-72), which is an ATP synthase. Alignment of similar proteins from other tick species showed the presence of the highly conserved dimerization motif (GxxxG) of the ATP synthase E chain (Figure S1), a transmembrane protein commonly associated with mitochondrial function. Its role in tick reproduction is uncertain. The 10.4 kDa protein reported in *A. hebraeum* matches the highly conserved acylphosphatases that are also found in diverse eukaryotic organisms including *I. scapularis* and contains the signature sequence for this enzyme (Figure S2). These hydrolases are important in glycolysis and gluconeogenesis; (4) *Guanine-nucleotide binding proteins* ( = G proteins). In mammals, G proteins are important signaling molecules on the cell surfaces in all cells and tissues. In the testis of mammals, G proteins are associated with the plasma membrane/outer acrosomal membrane region of acrosome-intact sperm. Fertilization requires opening of calcium ion channels in the sperm head which is regulated by G-proteins and serine/threonine phosphorylation [50]; this may explain the high frequency of serine/threonine kinase reads in the tick reproduction system (Table S1). A G protein (contig 1502) was identified in the male transcriptome as well as by LC MS/MS in the MAG/TVD (Table S2). Alignment of this protein with published sequences for *D. variabilis* and *Ornithodoros parkeri* shows high similarity including the structural tetrad of the WD40 conserved domain characteristic of this protein (Figure S3); equally noteworthy is upregulation of the message of a G-protein coupled receptor (GPCR) in the male accessory gland/testis/vas deferens (Table 1); (5) *Engorgement factor alpha* (Efα). Although the contig for Efα was found in our *D. variabilis* MAG/TVD transcriptome (Table S3), no evidence of the protein was found by LC MS/MS in the MAG/TVD or spermatophore (see above, section 3.3). In previous studies of mating in *D. variabilis*, gene silencing (RNAi) in males subsequently allowed to mate with females had no effect, i.e., there was no disruption of female engorgement to repletion or subsequent oviposition [19]. The Efα message was also ∼7 kb in size [19] as compared to the ∼800 bp message reported for Efα in *A. hebraeum* [3]. Efα in *D. variabilis* may have some role in male reproduction since the message only appears in fed males, but so far there is no evidence that the protein is acting as a male sex pheromone regulating female engorgement and reproduction; (6) *Ecdysone receptor*. A contig (03882) in the *D. variabilis* male transcriptome showed high identity (99%) to the tick ecdysone receptor. Alignment with other tick species, a spider and a scorpion showed the residues of the conserved ligand binding site, including the coactivator site common to the ecdysone receptors (Figure S4). Although the role of ecdysteroids, especially 20-hydroxyecdysone (20-E), in the biology of female ticks is well known [14], virtually nothing is known of its role in male reproduction. Consequently, the finding of the receptor for this hormone in the MAG/TVD may indicate an important signaling role for 20-E in male reproduction; (7) *Insect accessory gland (ACP) proteins*. No evidence of ACP proteins similar to those known in insects was found in the MAG/TVD transcriptome. BLAST searches (at the nucleotide level) were conducted using the reported *Drosophila* ACP sequences (including sex peptide ACP70) against the *D. variabilis* male transcriptome and the synganglion transcriptome [51]–[52] and against the *I. scapularis* genome. No significant matches were obtained against these databases (e-value cutoff of e^−^10); (8) *Subolesin.* In ticks, this molecule is involved in the regulation of gene expression including genes involved in multiple cellular pathways [53] and is known to affect blood digestion, development and reproduction. Recent RNAi studies also have shown that subolesin affects male reproduction and post-coital effects on mated females; female bont ticks (*A. hebraeum*) mated with conspecific males injected with subolesin dsRNA showed substantially higher mortality with survivors having substantially lower engorgement weights and smaller egg masses than controls. It was not clear from these studies whether the effects resulted from inhibition of an essential male stimulus or possibly as a result of insemination of the dsRNA construct by the treated males into the female during copulation [54]. Apparently the subolesin message is present in our *D. variabilis* MAG/TVD transcriptome (contig 12,359) with a percent similarity to that in *A. hebraeum* of 78% and an e value of 6.3e-15. The presence of the highly conserved GLICERMMKER region further supports this identification (Figure S5); (9) *Metalloproteases*. Although these enzymes are proteases, they are not usually involved in protein digestion; instead, they are typically associated with cell surfaces and sometimes are also secreted [13], [36]. Contig 00843 is a zinc metalloprotease with a very high match (6e-154, 70% identity) to a similar message found in the wasp, *Nasonia vitripennis*. A zinc-metalloprotease, similar to reproduction-associated fertilins and epidydimal proteins of the reprolysin family also was reported in *I. scapularis* [55]. Another contig from the *D. variabilis* MAG/TVD transcriptome (03261) is an astacin-like metalloproteinase with a match (4.4e-11) to an astacin-like metalloproteinase toxin from the spider, *Loxosceles intermedia*. Astacin and other metalloproteases have been found in male seminal fluids in *Drosophila*and are believed necessary to process male accessory gland proteins following insemination of the female [13]. Both of these contigs (00843 and 03261) in male *D. variabilis* MAG/TVD were significantly up-regulated in response to feeding (Table 1) suggesting a possible role in male reproduction; (10) *GPCR*. A contig for a G-protein coupled receptor (contig 08424) was identified in the male transcriptome that matched a GPCR in *I .scapularis* (e-value = 3.0 e-25). The qRT-PCR assay results showed that this receptor was significantly upregulated in fed versus unfed males (Table 1). Neurohormones, including neuropeptides and protein hormones “and their G protein-coupled receptors (GPCRs) play a central role in the control of behavior, reproduction, development, feeding and many other physiological processes” [56]. This G-protein coupled receptor in the male transcriptome closely matches a similar GPCR in the *I. scapularis* genome with a 432 bp region of the MAG/TVD message (contig 08424, Table S3) coding for a protein member of the Rhodopsin family. A BLAST match shows the closest similarity to a SIFamide receptor in the honeybee, *Apis mellifera* (NM_001113285; 3e-024). Studies in *Drosophila* showed that SIFamide regulated courtship behavior [57].

#### 3.4.2. Proteinases and proteinase inhibitors

Contigs for 6 different cysteine proteases and 12 different serine proteases/carboxypeptidases were identified in the MAG/TVD transcriptome (Tables S4 and S5). These are in addition to the 9 proteases, especially serine proteases (including trypsins) also found in the male reproductive tissues and spermatophore by LC MS/MS (Table S2). The high redundancy of serine protease and carboxypeptidase contigs is noteworthy and may attest to an important role in spermiogenesis and possibly post-coital stimulation of females. Note that both serine protease and trypsin were highly significantly upregulated in the fed male MAG/TVD (Table 1). The role of cysteine proteases in male reproductive activity, if any, is unknown. In the present study, expression of two cysteine proteases were examined: Cathepsin L (cysteine protease L, contig 05033) which was significantly down regulated in the male MAG/TVD following feeding and courtship and Cathepsin B (cysteine protease B, contig 00689) which was unchanged.

Contigs for 10 protease inhibitors were identified in the MAG/TVD transcriptome (Table S6). All are serpins (serine protease inhibitors). In locusts, as many as 5 different novel serpins were reported from ovaries and provide a novel form of regulatory control of oogenesis in these insects [58]. Whether they play a similar role in male tick reproduction is unknown.

#### 3.4.3. Amylases/hydrolases

Three contigs associated with carbohydrate digestion were identified (Table S7). These are in additional to the three carbohydrate digesting proteins found by LC MS/MS as described previously (section 3.3, Table S2).

#### 3.4.4. Lipases and lipid storage/mobilization

Ten contigs associated with lipid digestion or lipid storage were identified (Table S8). These are in addition to the three phospholipases found by LC MS/MS as described previously (section 3.3, Table S2). Phospholipases are key enzymes in the biosynthetic pathway leading to the synthesis of Prostaglandin E2. As shown in sandflies, lipases are important in reproductive activity having been found in the female accessory glands, ovarioles and oviducts [59]. Lipases also are known to be among the several important categories of insect accessory gland proteins (ACPs) [60]. In *Drosophila* spp., a high level of lipase activity was found in the male accessory glands prior to mating, and this lipase activity is transferred to the female during copulation [61]. However, no change in expression was found for phospholipase C by qRT-PCR in *D. variabilis* males (Table 1) even though the C isoform of this protein was found in the male accessory gland and spermatophore (Table S2).

#### 3.4.5. Oxidative and environmental stress

Protection against reactive oxygen species (ROS) is a critical cellular function for metabolically active tissues such as the male accessory glands and testis. Concomittantly, these proteins are also reported to protect against oxidative stress induced by microbial infections [62]. Nine contigs associated with oxidative stress and 4 associated with environmental stress were identified (Tables S9 and S10). These are in addition to the 9 oxidative stress proteins and 5 environmental stress proteins found by LC MS/MS as described previously (section 3.3, Table S2). Contigs for 2 copies of glutaredoxin (contigs 00433 and 00475) were identified. Alignment of contig 00433 with glutaredoxins from several insect species showed the 11 residues of the conserved ligand binding site and redox active motif in the 2 *D. variabilis* messages, suggesting a close similarity with sequences in GenBank (Figure S6 A). Similarly, contigs for 3 copies (contigs 05072, 09214 and 11946) of glutathione S-transferase (GST), including two that matched similar message in different tick species, were identified (Table S9). Alignment of contig 11946 with GSTs from *I. scapularis* and two mammalian species showed the presence of conserved domains (including the substrate binding pocket or H-site) (Figure S6 B). Other contigs tentatively identified as oxidative stress proteins include a glutamate dehydrogenase, oxidative stress induced growth inhibitor and thioredoxin. Contigs for glutaredoxin, GST (multiple copies), thioredoxin and other oxidative stress proteins were also found in the midgut of *D. variabilis* [62]. Analysis by qRT-PCR showed that thioredoxin was significantly upregulated in males in response to feeding and mating (Table 1).

The 4 contigs concerned with environmental stress (Table S10) include one methylmalonate semialdehyde dehydrogenase (MMSDH) and 3 heat shock proteins (in addition to the numerous HSPs identified by LC MS/MS). Alignment of contig 6257 with sequences from insects shows a very high level of agreement (including the cysteine residues of the catalytic sites), supporting its tentative identification as MMSDH (Figure S6 C). The 3 contigs for HSPs (contigs 00189, 12677 and 12744) are tentatively identified as such by virtue of their high e-values (1.5e-26, 5.6e-139 and 4.8e-73, respectively) and similarity to the HSPs previously identified by LC MS/MS (Table S2).

#### 3.4.6. Innate immunity

Seven contigs with putative functional assignments as innate immune peptides were identified (Table S11). These include a fibrinogen-related domain protein (FReD), lectins, a defensin, α-macroglobulin and a metalloproteinase. Alignment of contig 00983 with sequences from other ticks supports its identification as FReD, showing the conserved residues of the calcium binding site of the FReD domain and the polymerization pocket characteristic of these molecules (Figure S7 A). Alignment of contig 12360 with sequences from other ticks supports its tentative identification as a lectin with the FreD domain and a binding sites characteristic of innate immune peptides (Figure S7 B). Finally, alignment of contig 05417 with sequences from other tick species and presence of five of six conserved cysteines support its identification as a tick defensin (*varisin*) (Figure S7 C). However, several amino acid differences between this sequence and those of other ticks, including the conspecific *D. variabilis*, suggest that this contig may represent a different isoform. Another important species of immune peptides is basigin (contig 07749), an extracellular transmembrane matrix metalloproteinase and a member of the immunoglobulin superfamily. Basigin is related to neurothelin, CD147 and similar molecules that regulate membrane remodeling and cellular architecture in many organisms including insects such as *A. mellifera* and *D. melanogaster*. It is a cell surface receptor that has been shown to affect reproduction, e.g., mice deficient in the basigin gene are sterile [63]. Basigin also serves as a defense against pathological processes [64] (Table S11).

#### 3.4.7. Adhesion proteins

Ten contigs with putative functional assignments as cytoskeletal and/or adhesion were identified (Table S12). Adhesion-related proteins are of interest because of the possible related function of the sex peptide(s) that bind to sperm discussed previously [9]. Laminin-binding protein also is well represented in the MAG/TVD transcriptome of *D. variabilis*. Alignment of contig 12680 with sequences from other ticks and humans shows the RPS2s and S8 interaction sites and the putative laminin-1 binding site characteristic of these proteins (Figure S8 A). Another key adhesion molecule is tetraspanin. Tetraspanins are recognized as important components of the extracellular matrix proteins that contribute to the formation of cell to cell junctions [65]. Contigs for these proteins were also found in the MAG/TVD transcriptome (Table S12). Alignment of contig 10467 with sequences from other ticks and the human body louse shows the residues for the dimer interface on the conserved domain cd03127 and the tetraspanin large extracellular loop (Figure S8 B). Other binding proteins such as cadherins also mediate cell adhesion activity. A decline in the expression of cadherin has been shown to reduce spermatogenesis and germline stem cell maintenance in aging males of *Drosophila* [66].

### 4. Summary and future perspectives

In summary, this cDNA library of the fed/mating male reproductive system provides a transcriptome comprising 3898 expressed genes in 73 biological process categories (excluding 3,926 contigs that did not match any known sequence). Ten major GO categories were identified in this male reproductive cDNA library as follows: reproduction-related functions, peptidases, proteinase inhibitors, hydrolases, lipases, oxidative stress proteins, environmental stress proteins, innate immune proteins, cellular adhesion-cell surface modulating proteins and signal transduction receptors (GPCRs). In most cases, their functional assignments were supported by comparisons (BLAST) with sequences from other organisms, their low e-values, aligning the sequences with sequences from other species, and/or by changes in their expression level in unfed versus fed males of *D. variabilis*. In addition, proteins were also identified by LC MS/MS in the spermatophore and MAG/TVD; excluding metabolic, ribosomal and mitochondrial proteins, 42 of the 56 proteins identified by this method were found in the MAG/TVD transcriptome. Ef alpha but not Ef beta was found in our MAG/TVD transcriptome, suggesting that the regulation of female engorgement and the initiation of female reproduction by male pheromones is fundamentally different in *D. variabilis* than that described before for *A. hebraeum* [3].

The challenge for the future is to understand the physiology of male tick reproduction (an understudied area especially as compared to female ticks) and to understand the role of the proteins found in the spermatophore in the regulation of female reproduction. The question of how copulation stimulates female engorgement and reproduction is a black box for most ticks so far studied. Many possible leads for future research in this regard are presented in this paper. This catalogue of male reproductive transcripts may be useful for scientists investigating the molecular basis of reproduction in ticks as well as the search for candidate molecules for tick control.

## Materials and Methods

### 5.1. Ethics Statement

All use of animals for this research was carried out in strict accordance with the recommendations in the Guide for the Care and Use of Laboratory Animals of the Institute of Laboratory Animal Resources Commission on Life Sciences, National Research Council (http://www.nal.usda.gov/awic/pubs/noawicpubs/careuse.htm). The protocols were approved by the Old Dominion University Institutional Animal Care and Use Committee (IACUC). The approved protocols are on file in the Old Dominion University Office of Research Compliance (IACUC #10-017, 10-018 and 10-032).

### 5.2. Ticks

American dog ticks, *D. variabilis*, were reared as previously described [67] and originated from specimens collected near Richmond, Virginia, USA. Adult ticks were confined within plastic capsules attached to New Zealand white rabbits, *Oryctolagus cuniculus*, and allowed to feed as required. Larvae and nymphs were fed on Norway rats, *Rattus norvegicus*. Rearing conditions were 26±1°C, 92±1% relative humidity and 14∶10 Light versus dark (L∶ D).

### 5.3. 454 library preparation

Approximately 500 combined male accessory glands (MAG), testes and vas deferens (TVD) were dissected from *D. variabilis* males that were allowed to feed for 7–8 days without mating. Fed males were forcibly detached from their rabbit hosts, allowed to commence courtship behavior ( = premating) with *D. variabilis* females feeding on a different rabbit, and removed before they could inseminate the females (as determined by the absence of a spermatophore); males that copulated were discarded. Subsequently, the fed, female-exposed males were dissected; the male accessory glands, testes and vas deferens were excised; extraneous tissues removed; and the dissected male reproductive system washed twice in 4°C phosphate-buffered saline (PBS: pH 7.0, 10 mM NaH_2_PO_4_, 14 mM Na_2_HPO_4_, 150 mM NaCl). The cleaned tissues were homogenized in TRI Reagent according to the manufacturer's recommendations, and the subsequent RNA pellets were rehydrated in 100 µM aurin tricarboxylic acid to prevent degradation [68]. Samples were collected at different intervals and frozen until needed. The RNA from each sample was isolated and pooled. Approximately 4 µg of total RNA was obtained, and mRNA was isolated using an Oligotex mRNA isolation kit (Qiagen, Valencia, CA) according to the manufacturer's recommendations. Purified mRNA was ethanol precipitated, rehydrated in 2 µl of RNase-free water and combined with 10 pmol of modified 3′ reverse transcription primer (5′-ATTCTAGAGACCGAGGCGGCCGACATGT_(4)_GT_(9)_CT_(10)_VN-3′) [69] and 10 pmol SMART IV oligo (5′-AAGCAGTGGTATCAACGCAGAGTGGCCATTACGGCCGGG-3′) [70]. The resulting 4 µl were incubated at 72°C for 2 min and then combined with the following reagents on ice: 1 µl RNase Out (40 U/µl, 2 µl 5× first strand buffer, 1 µl 20 mM DTT, 1 µl dNTP mix (10 mM each) and 1 µl Superscript II reverse transcriptase) (Invitrogen, Carlsbad, CA). The reaction was incubated at 42°C for 90 min then diluted to 30 µl with TE buffer (10 mM Tris HCL pH 7.5, 1 mM EDTA) and stored at −20°C until further use. To synthesize second strand cDNA, 5 µl of first-strand cDNA was mixed with 10 pmol of modified 3′ PCR primer (5′-ATTCTAGAGGCCGAGGCGGCCGACATGT_(4)_GTCT_(4)_GTTCTGT_(3)_CT_(4)_VN-3′) [69], 10 pmol of 5′ PCR primer (5′-AAGCAGTGGTATCAACGCAGAGT-3′) [70], 5 µl 10× reaction buffer, 1 µl dNTP mix, 2 µl MgSO_4_, 0.4 µl Platinum HiFi Taq Polymerase and 34.6 µl H_2_O (Invitrogen). Thermal cycling conditions were 94°C for 2 min followed by 20 cycles of 94°C for 20 sec, 65°C for 20 sec and 68°C for 6 min. For optimization of the PCR reaction, 5 µl aliquots from cycles 18, 22 and 25 were analyzed on a 1% agarose gel. An additional 5 reactions were carried out with 20 cycles (the optimized number of cycles) to produce sufficient quantities of cDNA for preparation of the 454 library. The contents were combined, and the cDNA was purified using a PCR purification kit (Qiagen) according to the manufacturer's recommendations.

The cDNA library was prepared with the Standard Flex Platform kit (GS LR 70 sequencing kit, Cat. No. 04 932 315 001; Roche, Branford CT and Qiagen, Indianapolis, IN) for pyrosequencing on the GS-FLX sequencer (Roche) according to the manufacturer's recommendations which have described previously [71]. The only deviation from the protocol was that DNA-positive beads were enriched after emulsification PCR in order to increase the number of reads collected during titration. Enrichment was done so only beads containing DNA were loaded and the data generated during titration sequencing could also be used in the assembly of contiguous sequences. Enrichment of DNA-positive beads was completed exactly as described by Margulies et al. [71].

### 5.4. Quantitative real-time PCR (qRT-PCR)

Three samples, each consisting of 20–25 unfed or fed *D. variabilis* males were dissected. The combined male accessory glands, vas deferens and testis were excised, washed in PBS, extraneous tissues removed, and immediately homogenized in RLT buffer (Qiagen, Indianopolis, IN) and frozen on dry ice. DNase-treated (Qiagen) total RNA was isolated with the Qiagen RNAeasy kit according to the manufacturer's protocol. One microgram of total RNA from each sample was linearly amplified using the Invitrogen RT kit Invitrogen, Carlsbad, CA) and superscript II reverse transcriptase. The cDNA was diluted 1∶2 in nuclease-free H_2_O (Ambion, Austin, TX), and 2 µl were used per reaction for qRT-PCR with SYBR green master mix (BioRad, Foster City, CA). Reactions were conducted with a BioRad CFX96 Real Time instrument with iQ5 Optical system software (Version 2.1). Amplified products were normalized to Glyceraldehyde-3-Phosphate Dehydrogenase (GAPDH) (GB: EU999993) and analyzed using the 2^−Δ ΔC^ t method [72]. Primer sequences are shown in Table 2. Standard curves using serial dilutions of the GAPDH and selected gene primers were done according to the manufacture's recommendations to determine efficiency and reproducibility of the SYBR Green I assay (R^2^>0.97). Amplification specificity was confirmed by melting-curve analysis according to the manufacturer's recommendations. Comparisons of the qRT-PCR data for unfed versus fed male tissues were done using the PROC GLM procedure (SAS 9.1, SAS Institute, Cary, NC). Analysis of variance (ANOVA) was followed by pairwise comparisons of expression data using least squares means with a Tukey's adjustment in place to ensure an experiment-wise significance level of *P* = 0.05 for all comparisons.

### 5.5. Bioinformatics

Removal of primer sequence contamination and assembly of GS-FLX sequencing reads was carried out with the GS Assembler ver. 1.1.02.15 (Roche) using default parameters. Assembled contiguous sequences, herein referred to as contigs, were initially identified using the Tera-BLASTX algorithm with DeCypher (TimeLogic) against local custom databases consisting of the GenBank nr and EST databases (downloaded June 2008). BLAST searches [73] against the *Ixodes scapularis* genome and predicted transcripts were performed at the VectorBase website (www.vectorbase.org). Gene Ontology (GO) categorizations of the functional annotations of the top BLASTx hits (1e-10 cutoff) for biological processes (BP) levels 2 and 3 were done using the program Blast2GO [74], [75] in June 2010. Additional annotations were done as described by Pauchet et al. and Anderson et al. [62], [76] to provide a more extensive and detailed categorization of the relevant genes. Functional assignments were based on an e-10 cut-off (with selected exceptions as justified by other evidence) and conserved domain matches (SMART, KOG and Pfam databases). Clustal W was used for sequence alignments (www.ebi.ac.uk/clustalw/). Secretion signal prediction was carried out at the Signal P 3.0 server website (http://www.cbs.dtu.dk/services/SignalP) and searches were made using both neural networks and hidden Markov models [77]. BLAST searches [73] against the *I. scapularis* genomic contigs (ver. IscaW1) and predicted transcripts (ver. IscaW1.05.1) were performed at the Vectorbase website (www.vectorbase.org). Selected *Drosophila* sex peptides were used as queries against the GenBank nr and vectorbase databases.

### 5.6. Tissue collections, protein assays and gel electrophoresis

#### 5.6.1. Spermatophore collections

Partially-fed virgin females fed 6–7 days on a tick-naïve rabbit were forcibly detached and affixed by their mouthparts (capitulum) and front legs onto double-sided tape on a clear glass microscope slide. Males fed 7–8 days on a separate tick-naïve rabbit were forcibly detached and released adjacent to the taped females. Males that commenced mating with the females were observed under a stereoscopic microscope by inverting the glass slide, thereby enabling the observer to see the male with its mouthparts inserted into the female's genital pore. When a male withdrew its mouthparts, usually within less than 10 min, the male was removed with forceps to reveal the freshly deposited spermatophore inserted in the female's vulva. Each spermatophore was removed immediately and placed immediately in 20 µl of cold (4°C) protein collection buffer containing 150 mM PBS, 1∶200 diluted protease inhibitor cocktail (Sigma, St. Louis, MO) and 1∶50 lysis solution (0.1 g Tris base, 3.7 g EDTA, 0.5 g SDS, pH. 7.0). The spermatophore sample (total 20 spermatophores) was homogenized with an ultrasound dismembranator (Thermo Fisher Scientific, Suwanee, GA) for 10 sec pulse×2 while on wet ice and then frozen until ready for use. The protein concentration was determined using the Bradford protein assay using immunoglobulin G as a standard in accordance with the manufacturer's recommendations (BioRAD, Richmond, CA).

#### 5.6.2. Male accessory glands (MAG)/testis/vas deferens (TVD)

Fed males were retained and dissected following the commencement of courtship and/or copulation but before spermatophore insemination. MAG/TVD tissues were excised and placed into cold protein collection buffer as described by Donohue et al. [19] but with the addition of lysis buffer (described earlier). The samples were disrupted with a Kontes Model 749540 motorized hand-held plastic pestle (Kontes, Vineland, NJ), homogenized with a Virsonic ultrasound cell disruptor and probe (Virtis Co., Gardiner, NJ), centrifuged at 1,000× g for 10 min to remove particulates, and frozen (−80°C). Protein content was measured using the Bradford assay as described earlier.

#### 5.6.3. SDS-PAGE

Reducing gel electrophoresis was conducted using Tris-Bis 4–12% gradient NuPage minigels gels, 10 cm×10 cm×1 mm thick (Invitrogen, Carlsbad, CA). Samples of the spermatophore and MAG extracts were loaded (40 µg for the MAG extract versus 15 µg for the spermatophore extract) onto the gel. Gel electrophoresis was conducted under reducing conditions in accordance with the manufacturer's recommendations. Gels were stained with Coomassie Blue R and destained to reveal the protein bands. Relative molecular weights of the protein bands were estimated by comparison with molecular weight standards (Novex Sharp and Novex Mark 12, Invitrogen, Carlsbad, CA) and photographed with a digital camera. To compare differences in MAG protein expression, samples of the MAG extracts from unfed males and fed males exposed to females as described above were loaded (equal loading of 15 µg each) onto the protein gels and stained with silver stain (Invitrogen).

#### 5.6.4. Identification of selected protein bands from SDS-PAGE gels

Protein bands of interest (i.e., believed to contain proteins similar in size to those reported to be involved in reproductive activity) resolved from the spermatophore and MAG were excised from the gel, stored in distilled water and submitted to the W. M. Keck Biomedical Mass Spectrometry Laboratory (University of Virginia Health System, Charlottesville, VA) for protein identification by tryptic digestion and MS sequencing. The proteins in the gel bands were digested with trypsin, excess enzyme removed and analyzed using a Thermo Electron LTQFT mass spectrometer system interfaced with a Phenomenex Jupiter 10 µm C18 capillary column. The nanospray ion source was operated at 2.5 kV. The digest was analyzed using the double play capability of the instrument acquiring full scan mass spectra to determine peptide molecular weights and product ion spectra to determine amino acid sequence in sequential scans. The data were analyzed and proteins identified by database searching using the Sequest search algorithm against NCBI's NR database.

### Data Deposition

The Roche 454 reads of the *D. variabilis* male reproductive system transcriptome is registered with the NCBI Sequence Read Archive in the Transcriptome Shotgun Assembly (TSA) database under accession numbers JL943874–JL968929 (file 8_22_08_ nemaflytick_FFS9CHB04_ sub5.sqn).

## Supporting Information

Figure S1 **Multiple sequence alignments for ATP synthase from** ***D. variabilis*** **male transcriptome versus other species.** Multiple sequence alignment (ClustalW) of the deduced amino acid sequence of a putative *D. variabilis* ATP synthase (Contig1950) from the 454 transcriptome to the male reproductive system and other putative ATP synthases from ticks are compared. *Amblyomma americanum* (Aa; ACG76248), *Amblyomma hebraeum* (Ah; AF316621), *Ixodes scapularis* (Is; AAY66887), *Rhipicephalus sanguineus* (Rs; ACX53888). Light grey shading indicates the conserved residues of the dimerization motif (GxxxG) of ATP synthase E chain. Asterisks denote identical residues, dots indicate conservative substitutions.
(TIF)

Figure S2 **Multiple sequence alignments for acylphosphatase from** ***D. variabilis*** **male transcriptome versus other species.** Multiple sequence alignment (ClustalW) of the deduced amino acid sequence of a putative *D. variabilis* acylphosphatase (Contig 10624) identified from the 454 transcriptome to the male reproductive system and other putative and published acylphosphatases are compared. *Amblyomma hebraeum* (Ah; AAG45156), *Ixodes scapularis* (Is; EEC07914), *Pongo abelii* (Pa, XP_002812081), *Taeniopygia guttata* (Tg; ACH44949). The signature sequence for acylphosphatase 1 (PS00150) is shaded in light grey and the acylphosphatase 2 signature sequence (PS00151) is shaded in dark grey. Conserved catalytic Arg and Asn residues of acylphosphatase active sites are in black. Asterisks denote identical residues, dots indicate conservative substitutions.
(TIF)

Figure S3 **Multiple sequence alignments for guanine nucleotide protein from** ***D. variabilis*** **male transcriptome versus other species.** Multiple sequence alignment (ClustalW) of the deduced amino acid sequence of the 3′ region of a putative *D. variabilis* guanine nucleotide-binding protein (Contig 1502) identified from the 454 transcriptome to the male reproductive system and other guanine nucleotide-binding proteins are compared. *Dermacentor variabilis* (Dv; ACF35540), *Ixodes scapularis* (Is; AAY66933), *Ornithodoros parkeri* (Op; ABR23464). Eleven of the forty residues of the structural tetrad characteristic of the WD40 conserved domain are shaded. Asterisks denote identical residues, dots indicate conservative substitutions.
(TIF)

Figure S4 **Multiple sequence alignments for ecdysone receptor from** ***D. variabilis*** **male transcriptome versus other species.** Multiple sequence alignment (ClustalW) of the deduced amino acid sequence of a putative *D. variabilis* ecdysone receptor from the 454 transcriptome to the male reproductive system of *D. variabilis* with other ecdysone receptors from the Arthropoda are compared. *Dermacentor variabilis* (Dv; contig 3882), *Amblyomma americanum* (Aa1–3; ABB94566, ABB94567, ABB94565, respectively), *Agelena silvatica* (As; ADB24759), *Liocheles australasiae* (La; BAF85822). The twelve residues of the ligand binding domain that is conserved among ecdysone receptors are shaded in light grey. Residues of the putative coactivator site common to ecdysone receptors are shaded black. Asterisks denote identical residues, dots indicate conservative substitutions.
(TIF)

Figure S5 **Multiple sequence alignments for subolesin from** ***D. variabilis*** **male transcriptome versus other species.** Multiple sequence alignment (ClustalW) of the deduced amino acid sequence of a putative *D. variabilis* subolesin from the *D. variabilis* male reproductive system transcriptome with other putative tick subolesins are compared. *Dermacentor variabilis* (Contig 12359), *Amblyomma americanum* (Aa; ABA62326), *Amblyomma hebraeum* (Ah; ABY84524), *Dermacentor marginatus* (Dm; ABA62333), *Dermacentor variabilis* (Dv; AAV67034), *Haemaphysalis punctata* (Hp; ABA62336). No conserved domain has been reported for this molecule. The 11-mer highly conserved site GLICERMMKER is underlined and in bold. Asterisks denote identical residues, dots indicate conservative substitutions.
(TIF)

Figure S6 **Multiple sequence alignments for oxidative and environmental stress proteins from** ***D. variabilis*** **male transcriptome versus other species.** Multiple sequence alignments (ClustalW) of the deduced amino acid sequences of contigs from the *D. variabilis* male reproductive system transcriptome with other putative genes from the Arthropoda putatively involved in oxidative and environmental stress. **A**. Glutaredoxin; *Dermacentor variabilis* (Contig 433), *Drosophila virilis* (Dv; XP_002047597), *Pediculus humanus corporis* (Phc; XP_002430689), *Saccoglossus kowalevskii* (Sk; XP_002730955), *Tribolium castaneum* (Tc; XP_975253), *Tetraodon nigroviridis* (Tn; CAG03692). The eleven residues of the glutathione binding site that is conserved among glutaredoxins are shaded in light grey. The redox active CXXC motif is underlined and bolded. **B**. Glutathione S-transferase (GST); *Dermacentor variabilis* (Contig11946), *Bos taurus* (Bt; AAI42537), *Ixodes scapularis* (Is; XM_002401353), *Monodelphis domestica* (Md; XP_001381960). The soluble GST N-terminal domain profile is in bold (PROSITE). Dark grey shading denotes three of the four residues of the dimer interface on the GST_C_Mu conserved domain. Five of the seven residues of the substrate binding pocket (H-site) on the GST_C_Mu conserved domain are shaded in light grey. Two of seven residues of the N-terminal domain interface on the GST_C_Mu conserved domain are underlined. **C**. Methylmalonate semialdehyde dehydrogenase (MMSDH); *Dermacentor variabilis* (Contig6257), *Anopheles gambiae* (Ag; XP_312441), *Culex quinquefasciatus* (Cq; XP_001853240), *Pediculus humanus corporis* (Phc; XP_002425933). The Cys residue shaded in dark grey is one of the four catalytic residues of the MMSDH conserved domain (cd07085). Residues of the tetrameric interface of cd07085 are shaded in light grey. Four of the 24 residues of the NAD(P) binding site of cd07085 are in bold. Asterisks denote identical residues; conservative substitutions are indicated by two dots.
(TIF)

Figure S7 **Multiple sequence alignments for innate immune proteins from** ***D. variabilis*** **male transcriptome versus other species.** Multiple sequence alignments (ClustalW) of the deduced amino acid sequences of contigs from the *D. variabilis* male reproductive system transcriptome with other putative genes from the Arthropoda putatively involved in innate immunity are compared. **A**. Fibrinogen-related protein (FReD) (ixoderin-like peptides) ( = lectins); *Dermacentor variabilis* (Contig 00983) *Hyalomma marginatum rufipes* (Hmr; ADN23533), *Ixodes ricinus* (Ir; AAG93650), *Ixodes scapularis* (Is; XM_002411674), *Ornithodoros moubata* (Om; AAM88421). Light grey shading denotes the three conserved residues of the calcium binding site of fibrinogen-related domains (FReDs). Dark grey shading shows residues of the polymerization pocket of FReDs. **B**. Lectin; *Dermacentor variabilis* (Contig 12360), *Ixodes ricinus* (Ir; AY341424), *Ixodes scapularis* (Is; XM_002411674), *Rhipicephalus sanguineus* (Rs; EF490692). Light grey shading shows one of the characteristic lectin domains. **C**. Defensin; *Dermacentor variabilis* (Contig5417), *Dermacentor marginatus* (Dm; ACJ04433), *Dermacentor variabilis* (Dv; AY181027), *Haemaphysalis longicornis* (Hl; BAD93183), *Ixodes scapularis* (Is; XP_002436104), *Rhipicephalus microplus* (Rm; AY233213). Cys residues putatively involved in the formation of disulfide bonds are shaded light grey. Residues in the contig sequence that are different from all other sequences are shaded in dark grey. Asterisks indicate identical residues, dots are conservative substitutions.
(TIF)

Figure S8 **Multiple sequence alignments for adhesion proteins from** ***D. variabilis*** **male transcriptome versus other species.** Multiple sequence alignments (ClustalW) of the deduced amino acid sequences of contigs from the *D. variabilis* male reproductive system transcriptome with putative adhesion proteins from the Arthropoda are compared. **A**. Ribosomal protein S2 (RPS2) containing a laminin-1 binding protein domain; *Dermacentor variabilis* (Contig12680), *Hyalomma marginatum rufipes* (Hm; ADN23554), *Homo sapiens* (Hs; AAP36925), *Ornithodoros parkeri* (Op A6NA00). Eight of the thirteen residues of the rRNA interaction site of RPS2s are shaded in light grey. The seven residues of the conserved S8 interaction site are in bold. The six residues of the putative laminin-1 binding site are shaded dark grey. **B**. Tetraspanin; *Dermacentor variabilis* (Contig10467), *Dermacentor variabilis* (Dv; AAL75584), *Ixodes scapularis* (Is; AAY66979), *Pediculus humanus corporis* (Phc; XP_002429537). Light grey shading indicates the conserved residues of the dimer interface on the conserved domain cd03127: tetraspanin large extracellular loop. Asterisks indicate identical residues, dots are conservative substitutions.
(TIF)

Table S1 **The 50 most abundant contigs from the** ***D. variabilis*** **male accessory gland/testis vas deferens transcriptome^1^.**
(DOCX)

Table S2 **Comparison of contigs from the male transcriptome identified by BLASTx with their corresponding proteins/peptides in the MAG/TVD and/or spermatophore identified by LC/MS/MS (excluding common house-keeping proteins).^1,2^**
(DOCX)

Table S3 **Contigs in** ***D. variabilis*** **fed male accessory glands/testis/vas deferens associated with reproductive activity.**
(DOCX)

Table S4 **Contigs in** ***D. variabilis*** **fed male accessory glands/testis/vas deferens associated with protein digestion by cysteine proteinases.**
(DOCX)

Table S5 **Contigs in** ***D. variabilis*** **fed male accessory glands/testis/vas deferens associated with protein digestion by serine proteinases/carboxypeptidases.**
(DOCX)

Table S6 **Contigs in** ***D. variabilis*** **fed male accessory glands/testis/vas deferens associated with control of reproductive activity by proteinase inhibitors.**
(DOCX)

Table S7 **Contigs in** ***D. variabilis*** **fed male accessory glands/testis/vas deferens associated with control of carbohydrate digestion by amylases/hydrolases.**
(DOCX)

Table S8 **Contigs in** ***D. variabilis*** **fed male accessory glands/testis/vas deferens associated with control of lipid digestion/lipid storage by lipases.**
(DOCX)

Table S9 **Contigs in** ***D. variabilis*** **fed male accessory glands/testis/vas deferens associated with oxidative stress.**
(DOCX)

Table S10 **Contigs in** ***D. variabilis*** **fed male accessory glands/testis/vas deferens associated with environmental stress.**
(DOCX)

Table S11 **Contigs in** ***D. variabilis*** **fed male accessory glands/testis/vas deferens associated with innate immunity.**
(DOCX)

Table S12 **Contigs in** ***D. variabilis*** **fed male accessory glands/testis/vas deferens associated with cytoskeletal/cell adhesion.**
(DOCX)
