# Supplementary figures and images for: First Transcriptome of the Testis-Vas Deferens-Male Accessory Gland and Proteome of the Spermatophore from Dermacentor variabilis (Acari: Ixodidae)
Source: PLoS One. 2011 Sep 16;6(9):e24711. doi: 10.1371/journal.pone.0024711 (PMC3174968; doi:10.1371/journal.pone.0024711)

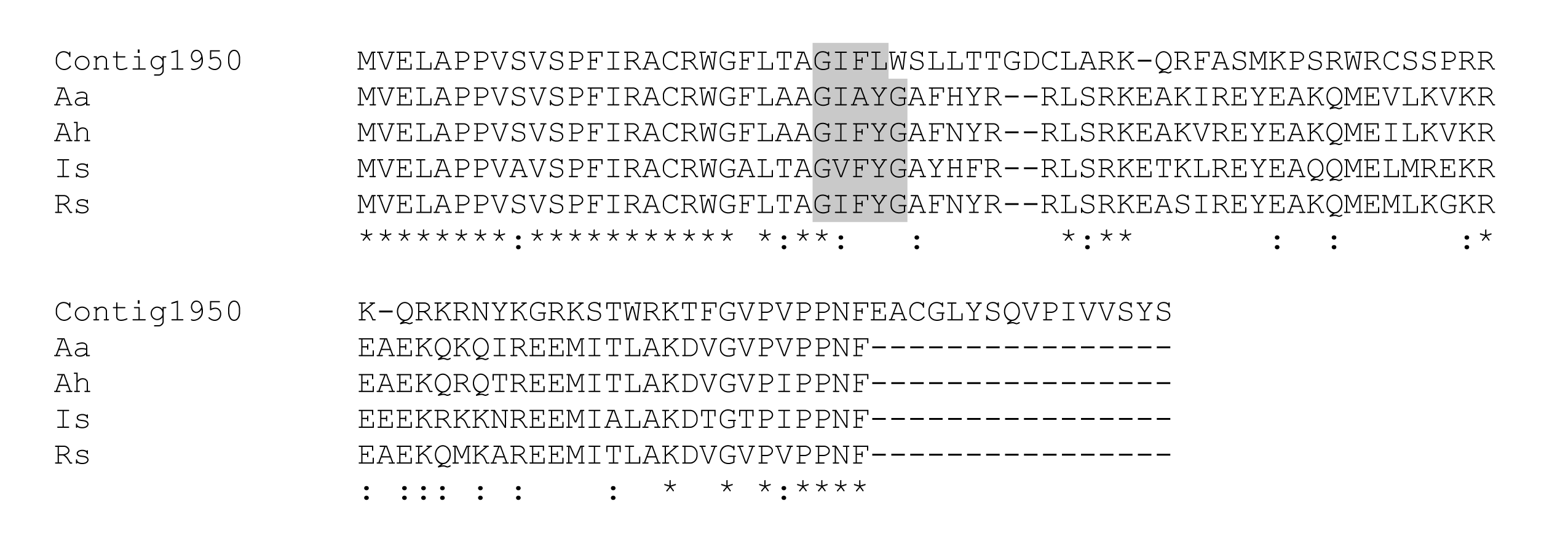

Supplement: Figure S1 — Multiple sequence alignments for ATP synthase from D. variabilis male transcriptome versus other species. Multiple sequence alignment (ClustalW) of the deduced amino acid sequence of a putative D. variabilis ATP synthase (Contig1950) from the 454 transcriptome to the male reproductive system and other putative ATP synthases from ticks are compared. Amblyomma americanum (Aa; ACG76248), Amblyomma hebraeum (Ah; AF316621), Ixodes scapularis (Is; AAY66887), Rhipicephalus sanguineus (Rs; ACX53888). Light grey shading indicates the conserved residues of the dimerization motif (GxxxG) of ATP synthase E chain. Asterisks denote identical residues, dots indicate conservative substitutions. (TIF) [file pone.0024711.s001.tif]

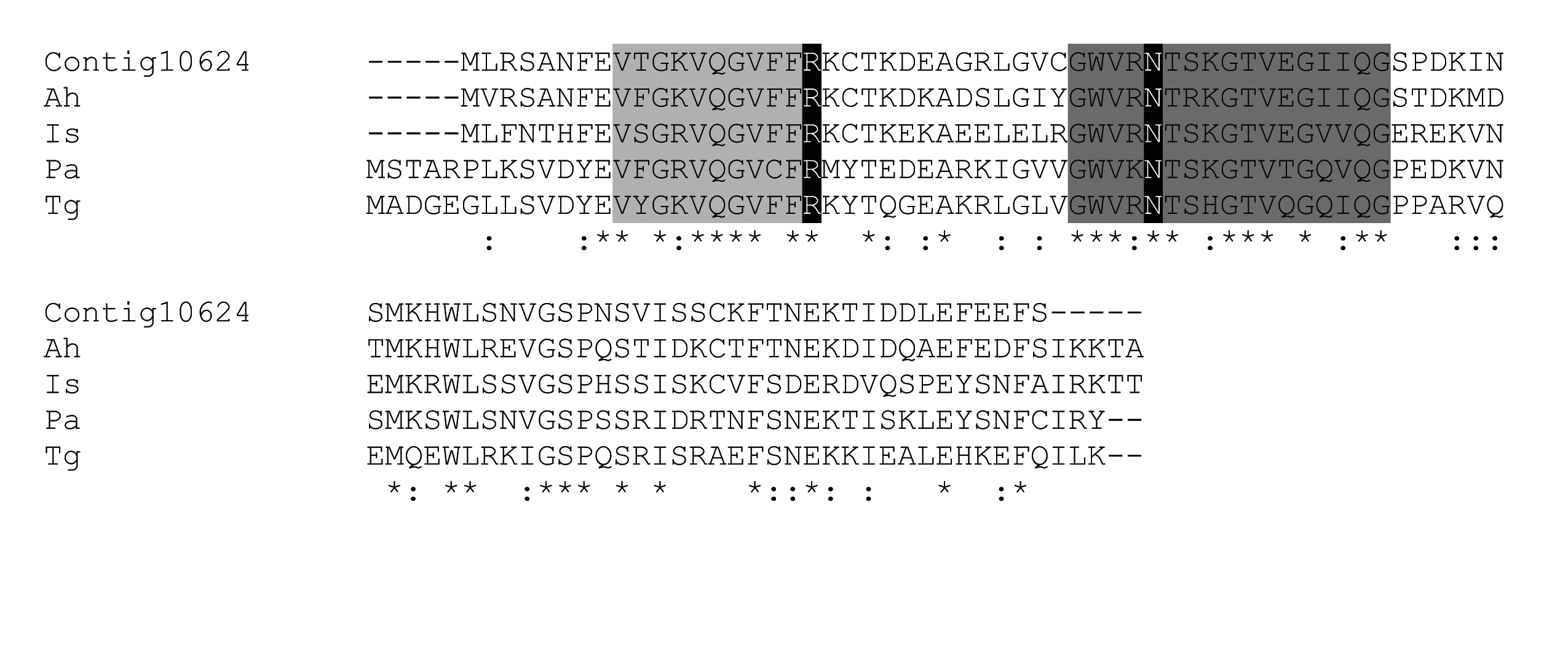

Supplement: Figure S2 — Multiple sequence alignments for acylphosphatase from D. variabilis male transcriptome versus other species. Multiple sequence alignment (ClustalW) of the deduced amino acid sequence of a putative D. variabilis acylphosphatase (Contig 10624) identified from the 454 transcriptome to the male reproductive system and other putative and published acylphosphatases are compared. Amblyomma hebraeum (Ah; AAG45156), Ixodes scapularis (Is; EEC07914), Pongo abelii (Pa, XP_002812081), Taeniopygia guttata (Tg; ACH44949). The signature sequence for acylphosphatase 1 (PS00150) is shaded in light grey and the acylphosphatase 2 signature sequence (PS00151) is shaded in dark grey. Conserved catalytic Arg and Asn residues of acylphosphatase active sites are in black. Asterisks denote identical residues, dots indicate conservative substitutions. (TIF) [file pone.0024711.s002.tif]

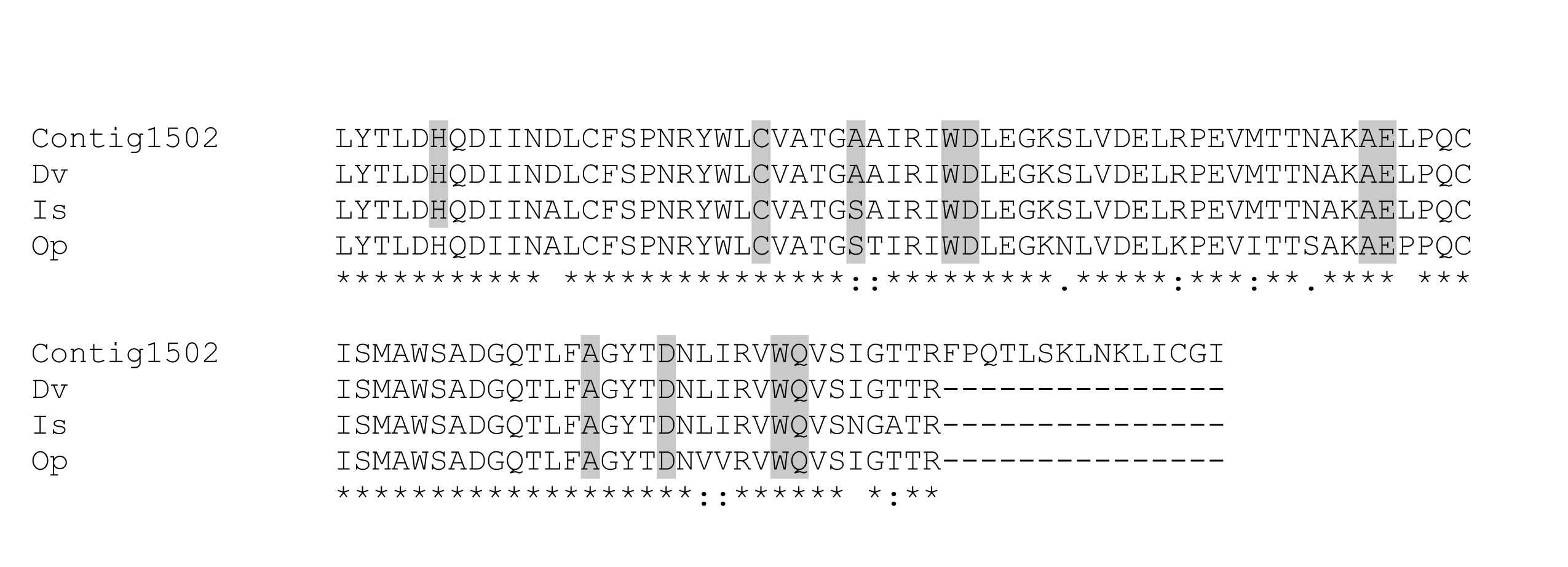

Supplement: Figure S3 — Multiple sequence alignments for guanine nucleotide protein from D. variabilis male transcriptome versus other species. Multiple sequence alignment (ClustalW) of the deduced amino acid sequence of the 3′ region of a putative D. variabilis guanine nucleotide-binding protein (Contig 1502) identified from the 454 transcriptome to the male reproductive system and other guanine nucleotide-binding proteins are compared. Dermacentor variabilis (Dv; ACF35540), Ixodes scapularis (Is; AAY66933), Ornithodoros parkeri (Op; ABR23464). Eleven of the forty residues of the structural tetrad characteristic of the WD40 conserved domain are shaded. Asterisks denote identical residues, dots indicate conservative substitutions. (TIF) [file pone.0024711.s003.tif]

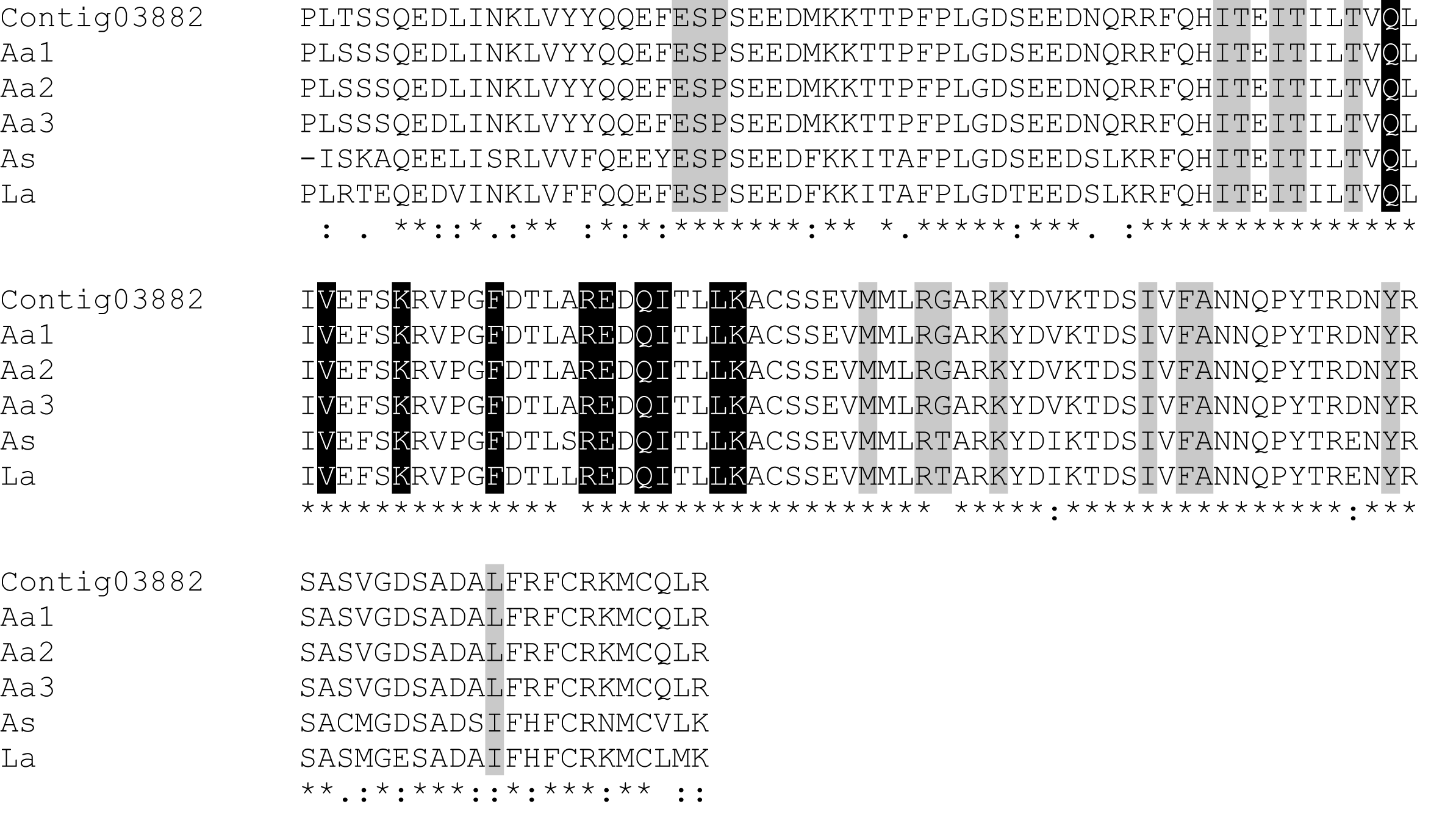

Supplement: Figure S4 — Multiple sequence alignments for ecdysone receptor from D. variabilis male transcriptome versus other species. Multiple sequence alignment (ClustalW) of the deduced amino acid sequence of a putative D. variabilis ecdysone receptor from the 454 transcriptome to the male reproductive system of D. variabilis with other ecdysone receptors from the Arthropoda are compared. Dermacentor variabilis (Dv; contig 3882), Amblyomma americanum (Aa1–3; ABB94566, ABB94567, ABB94565, respectively), Agelena silvatica (As; ADB24759), Liocheles australasiae (La; BAF85822). The twelve residues of the ligand binding domain that is conserved among ecdysone receptors are shaded in light grey. Residues of the putative coactivator site common to ecdysone receptors are shaded black. Asterisks denote identical residues, dots indicate conservative substitutions. (TIF) [file pone.0024711.s004.tif]

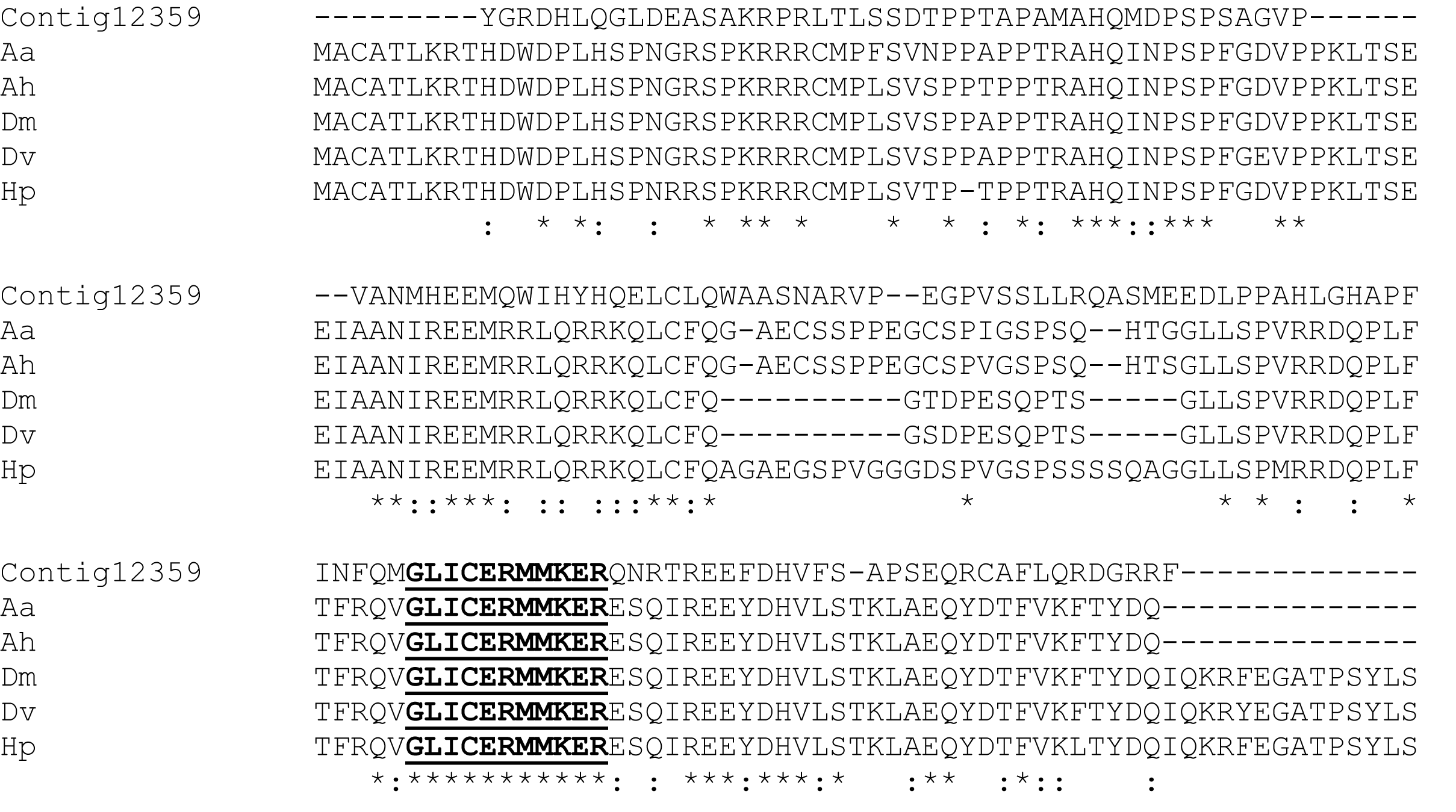

Supplement: Figure S5 — Multiple sequence alignments for subolesin from D. variabilis male transcriptome versus other species. Multiple sequence alignment (ClustalW) of the deduced amino acid sequence of a putative D. variabilis subolesin from the D. variabilis male reproductive system transcriptome with other putative tick subolesins are compared. Dermacentor variabilis (Contig 12359), Amblyomma americanum (Aa; ABA62326), Amblyomma hebraeum (Ah; ABY84524), Dermacentor marginatus (Dm; ABA62333), Dermacentor variabilis (Dv; AAV67034), Haemaphysalis punctata (Hp; ABA62336). No conserved domain has been reported for this molecule. The 11-mer highly conserved site GLICERMMKER is underlined and in bold. Asterisks denote identical residues, dots indicate conservative substitutions. (TIF) [file pone.0024711.s005.tif]

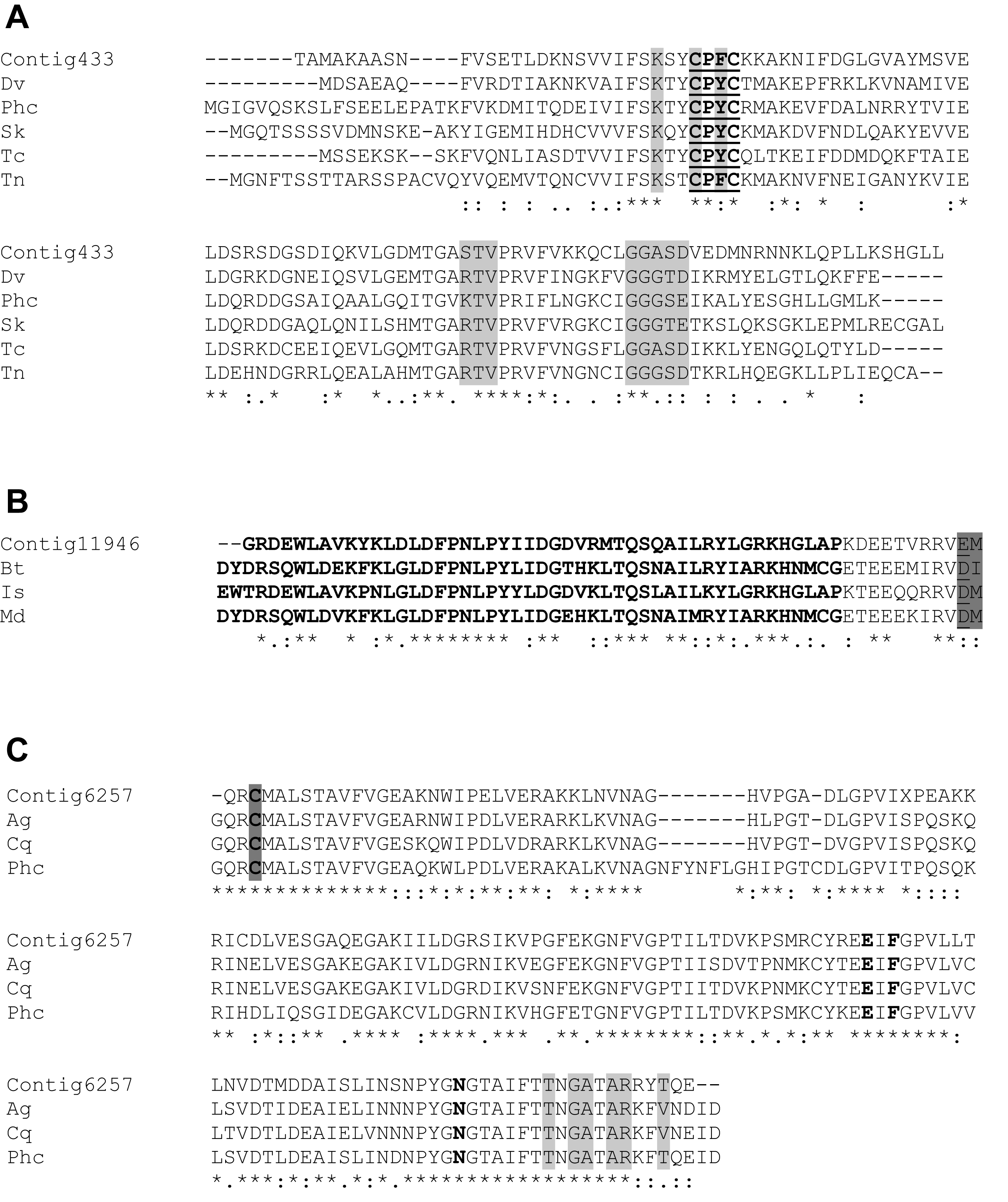

Supplement: Figure S6 — Multiple sequence alignments for oxidative and environmental stress proteins from D. variabilis male transcriptome versus other species. Multiple sequence alignments (ClustalW) of the deduced amino acid sequences of contigs from the D. variabilis male reproductive system transcriptome with other putative genes from the Arthropoda putatively involved in oxidative and environmental stress. A. Glutaredoxin; Dermacentor variabilis (Contig 433), Drosophila virilis (Dv; XP_002047597), Pediculus humanus corporis (Phc; XP_002430689), Saccoglossus kowalevskii (Sk; XP_002730955), Tribolium castaneum (Tc; XP_975253), Tetraodon nigroviridis (Tn; CAG03692). The eleven residues of the glutathione binding site that is conserved among glutaredoxins are shaded in light grey. The redox active CXXC motif is underlined and bolded. B. Glutathione S-transferase (GST); Dermacentor variabilis (Contig11946), Bos taurus (Bt; AAI42537), Ixodes scapularis (Is; XM_002401353), Monodelphis domestica (Md; XP_001381960). The soluble GST N-terminal domain profile is in bold (PROSITE). Dark grey shading denotes three of the four residues of the dimer interface on the GST_C_Mu conserved domain. Five of the seven residues of the substrate binding pocket (H-site) on the GST_C_Mu conserved domain are shaded in light grey. Two of seven residues of the N-terminal domain interface on the GST_C_Mu conserved domain are underlined. C. Methylmalonate semialdehyde dehydrogenase (MMSDH); Dermacentor variabilis (Contig6257), Anopheles gambiae (Ag; XP_312441), Culex quinquefasciatus (Cq; XP_001853240), Pediculus humanus corporis (Phc; XP_002425933). The Cys residue shaded in dark grey is one of the four catalytic residues of the MMSDH conserved domain (cd07085). Residues of the tetrameric interface of cd07085 are shaded in light grey. Four of the 24 residues of the NAD(P) binding site of cd07085 are in bold. Asterisks denote identical residues; conservative substitutions are indicated by two dots. (TIF) [file pone.0024711.s006.tif]

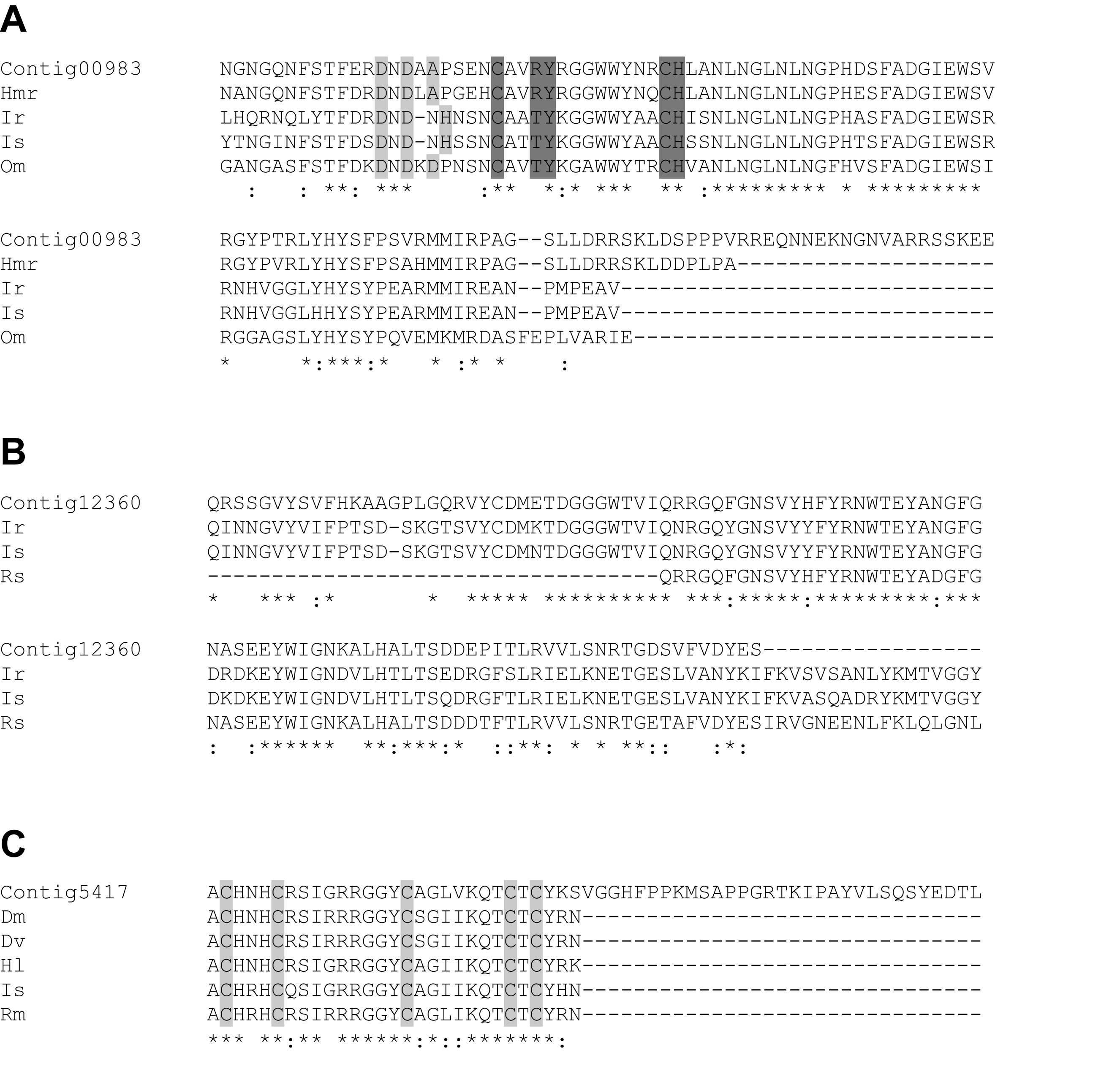

Supplement: Figure S7 — Multiple sequence alignments for innate immune proteins from D. variabilis male transcriptome versus other species. Multiple sequence alignments (ClustalW) of the deduced amino acid sequences of contigs from the D. variabilis male reproductive system transcriptome with other putative genes from the Arthropoda putatively involved in innate immunity are compared. A. Fibrinogen-related protein (FReD) (ixoderin-like peptides) ( = lectins); Dermacentor variabilis (Contig 00983) Hyalomma marginatum rufipes (Hmr; ADN23533), Ixodes ricinus (Ir; AAG93650), Ixodes scapularis (Is; XM_002411674), Ornithodoros moubata (Om; AAM88421). Light grey shading denotes the three conserved residues of the calcium binding site of fibrinogen-related domains (FReDs). Dark grey shading shows residues of the polymerization pocket of FReDs. B. Lectin; Dermacentor variabilis (Contig 12360), Ixodes ricinus (Ir; AY341424), Ixodes scapularis (Is; XM_002411674), Rhipicephalus sanguineus (Rs; EF490692). Light grey shading shows one of the characteristic lectin domains. C. Defensin; Dermacentor variabilis (Contig5417), Dermacentor marginatus (Dm; ACJ04433), Dermacentor variabilis (Dv; AY181027), Haemaphysalis longicornis (Hl; BAD93183), Ixodes scapularis (Is; XP_002436104), Rhipicephalus microplus (Rm; AY233213). Cys residues putatively involved in the formation of disulfide bonds are shaded light grey. Residues in the contig sequence that are different from all other sequences are shaded in dark grey. Asterisks indicate identical residues, dots are conservative substitutions. (TIF) [file pone.0024711.s007.tif]

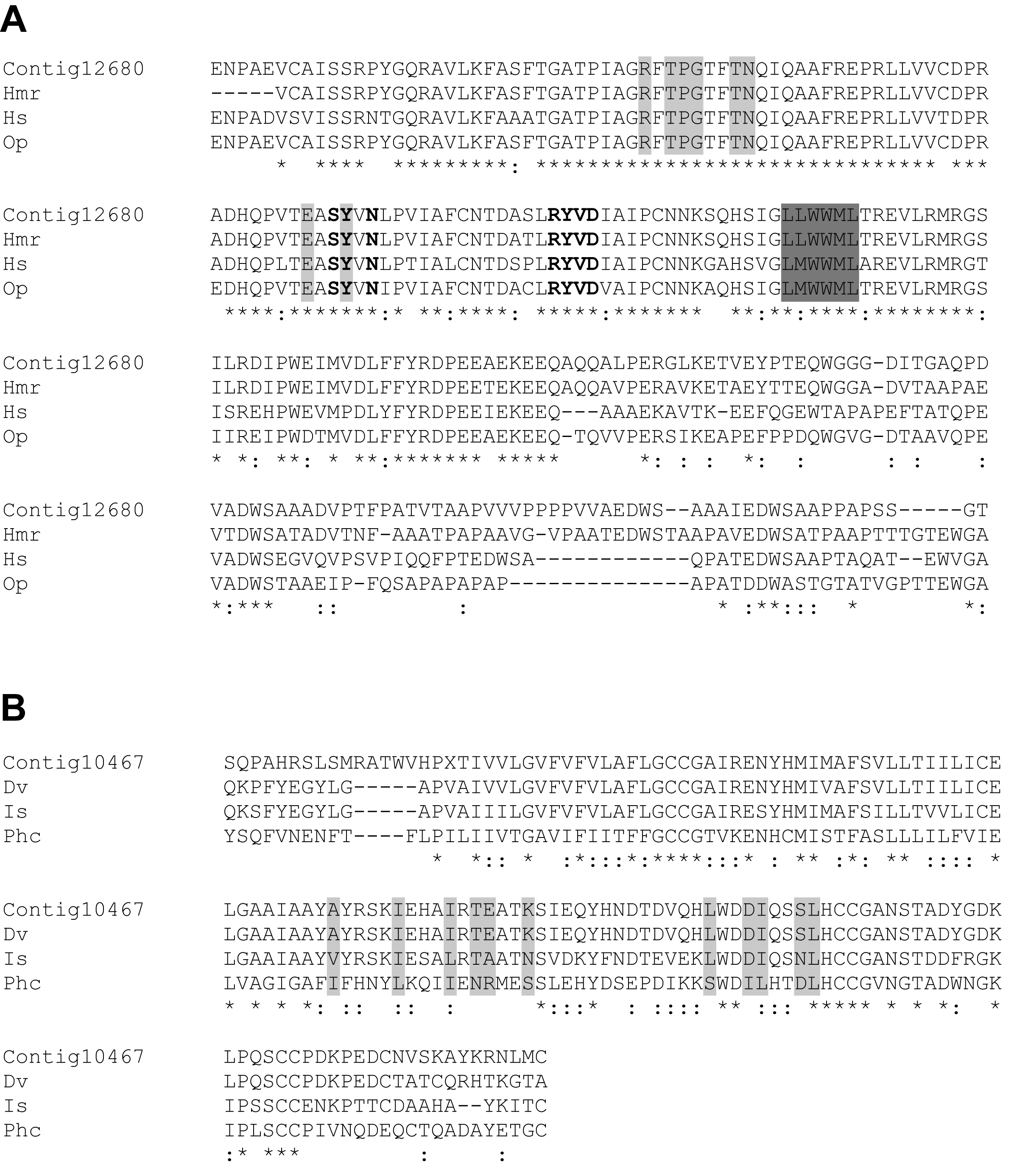

Supplement: Figure S8 — Multiple sequence alignments for adhesion proteins from D. variabilis male transcriptome versus other species. Multiple sequence alignments (ClustalW) of the deduced amino acid sequences of contigs from the D. variabilis male reproductive system transcriptome with putative adhesion proteins from the Arthropoda are compared. A. Ribosomal protein S2 (RPS2) containing a laminin-1 binding protein domain; Dermacentor variabilis (Contig12680), Hyalomma marginatum rufipes (Hm; ADN23554), Homo sapiens (Hs; AAP36925), Ornithodoros parkeri (Op A6NA00). Eight of the thirteen residues of the rRNA interaction site of RPS2s are shaded in light grey. The seven residues of the conserved S8 interaction site are in bold. The six residues of the putative laminin-1 binding site are shaded dark grey. B. Tetraspanin; Dermacentor variabilis (Contig10467), Dermacentor variabilis (Dv; AAL75584), Ixodes scapularis (Is; AAY66979), Pediculus humanus corporis (Phc; XP_002429537). Light grey shading indicates the conserved residues of the dimer interface on the conserved domain cd03127: tetraspanin large extracellular loop. Asterisks indicate identical residues, dots are conservative substitutions. (TIF) [file pone.0024711.s008.tif]
