# Supplementary material for: First Transcriptome of the Testis-Vas Deferens-Male Accessory Gland and Proteome of the Spermatophore from Dermacentor variabilis (Acari: Ixodidae)
Source: PLoS One. 2011 Sep 16;6(9):e24711. doi: 10.1371/journal.pone.0024711 (PMC3174968; doi:10.1371/journal.pone.0024711)
Supplement: Table S1 — The 50 most abundant contigs from the D. variabilis male accessory gland/testis vas deferens transcriptome1. (DOCX) [file pone.0024711.s009.docx]

Table S1. The 50 most abundant contigs from the *D. variabilis* male accessory gland/testis vas deferens transcriptome.^1^

| **Contig** | **Length^2^** | **Reads** | **Best Match to NR Protein database^3^** | **Accession No.** | **E value** | **Conserved**  **Domains^4^** | **Putative function** |
| --- | --- | --- | --- | --- | --- | --- | --- |
| 12738 | 161 | 3410 | Similar to CG14305-PA, *A. mellifera* | XP_394567 | 9.4e-12 | None | Serine/Threonine protein kinases, |
| 12106 | 325 | 3126 | ferritin, *D. variabilis* | AAL75582 | 6.3e-38 | None | Stores iron in soluble, non-toxic, form |
| 12696 | 128 | 2135 | serine/threonine kinase 22B, *H. sapiens* | NP_443732 | 4.8e-3 | None | Testis/spermiogenesis-specific kinase – **in MAG** |
| 12544 | 586 | 1920 | retinol dehydrogenase, *X. laevis* | NP_001090337 | 5.5e-16 | None | Housekeeping: Regulates synthesis retinoic acid |
| 12619 | 108 | 1843 | Ferritin, *D. variabilis* | AAL75582 | 5.e-13 | None | Stores iron in soluble, non-toxic, form |
| 12201 | 418 | 1711 | 60s ribosomal protein, *L. singoriensis* | ABX75387 | 7.6e-27 | None | Housekeeping: Protein binding |
| 12448 | 381 | 1620 | 40 S ribosomal protein, *O. parkeri* | ABR23354 | 7.1e-60 | Pfam 03719 | Housekeeping: laminin surface receptor; cell adhesion to the basement membrane; cell signaling. |
| 11464 | 2506 | 1596 | Metalloendopeptidase, *A. mellifera* | XP_001122158 | 1.9e-17 | None | Post-translational modification, protein turnover |
| 12335 | 413 | 1578 | myosin essential light chain, *I. scapularis* | AAY66877 | 1.3e-71 | None | Interacting with myosin I complex |
| 12422 | 479 | 1520 | Hexokinase A, *D. santomea* | AAV91306 | 1.6e-44 | None | glucose ATP phosphotransferase activity |
| 12324 | 170 | 1502 | Cytochrome C oxidase, *A. monolakensis* | ABI52811 | 2.9e-21 | None | Electron transport, respiratory metabolism |
| 10561 | 767 | 1307 | Nucleoporin, *N. vitripennis* | XP_00160520 | 7.4e-11 | Pfam 8911 | Transport of proteins through nuclear membrane |
| 11512 | 236 | 1246 | Neprilysin, *D. melanogaster* | NP_649924 | 1.0e-04 | COG3590 | Metalloprotease involved in sperm function and fertilization **– in spermatophore** |
| 12677 | 1583 | 1180 | Heat shock protein HSP 70, *H. sapiens* | NP_006588 | 5.6e-139 | PTZ00009 | Stress response, intracellular chaperones |
| 12640 | 318 | 1134 | ubiquitin conjugating enzyme, *B. mori* | NP_001103772 | 2.0e-28 | None | Protein degradation |
| 12193 | 632 | 993 | Glyoxylate reductase, *H. sapiens* | EAW58286 | 4.8e-38 | None | Carbohydrate metabolism |
| 00022 | 1621 | 983 | Proteophosphoglycan, *L. major* | XP_843164 | 1.0e-66 | None | Causes vacuolization of peritoneal macrophages |
| 12716 | 1100 | 924 | Villin 1, *X. tropicalis* | NP_001005657 | 4.7E-40 | None | Regulates actin length and structure |
| 10477 | 1371 | 923 | Helicase, *S. purpuratus* | XP_783941 | 2.4e-71 | COG0553 | Transcription/DNA replication; unwind nucleic acid duplexes with a distinct directional polarity |
| 12389 | 377 | 911 | Keratin, *O. cuniculus* | Q29426 | 2.1e-69 | None | Cytoskeletal structure of cells |
| 12779 | 149 | 909 | Nucleotide excision repair, *A. melifera* | XP_623093 | 7.3e-12 | None | DNA repair mechanism |
| 12257 | 516 | 893 | ubiquitin-conjugating enzyme, *N. vitripennis* | XP_001608299 | 4.8e-83 | None | Intracellular protein degradation, protein turnover. |
| 12464 | 1227 | 890 | Ca2+-binding protein, *T. infestans* | XP_001601434 | 1.5e-48 | COG5126 | Calcium signal modulators; apoptosis inducing gene |
| 12428 | 219 | 837 | ubiquitin conjugating enzyme, *B. mori* | NP_001103772 | 7.9e-37 | None | Protein degradation |
| 12356 | 199 | 823 | Serine protease, *Ae. aegypti* | XP_001655706 | 8.2e-04 | None | Proteolysis; protein digestion **– in spermatophore** |
| 12759 | 164 | 820 | 14-3-3 CG17870-PA, *A. mellifera* | XP_623183 | 1.4e-23 | None | Regulate cell/protein signaling activity |
| 12307 | 194 | 777 | Transmembrane protein, *R. solanacearum* | NP_521951 | 0.323 | None | Cell motility; sperm migration **– in MAG** |
| 10841 | 676 | 776 | Endothelin-converting enzyme, *H. sapiens* | NP_001106818 | 2.7e-08 | None | Regulation vessel constriction; protein degradation. |
| 11358 | 529 | 770 | Engorgement factor alpha, *D. variabilis* | ABM92922.1 | 1.1e-49 | None | Part of proteasome |
| 12015 | 467 | 764 | *I. scapularis* hypothetical protein | NP_001068612 | 2.0e-14 | None | Unknown |
| 11565 | 377 | 760 | Nucleoporin, *B. taurus* | NP_001033137 | 4.6e-27 | Cd00835 | Regulate protein flow across nuclear membrane |
| 11351 | 1219 | 736 | Elongation factor 1-alpha, *Amblyomma* spp | AAK12647 | 1.2e-167 | Cl03705 | Protein translation in ribosomes; protein biosynthesis; part of proteasome |
| 11668 | 2015 | 725 | Collagen binding protein, *T. casteneum* | XP_970605 | 2.0e-123 | Pfam01852 | Collagen binding; lipid binding and transport |
| 11880 | 607 | 721 | ubiquitin conjugating enzyme, *H. sapiens* | AAH15169 | 4.9e-10 | Cl00195 | Intracellular protein degradation, protein turnover |
| 12749 | 1413 | 718 | Serine/threonine kinase, *H. sapiens* | AAH65499 | 1.2e-04 | Cd00987 | Protein phosphorylation- **in MAG** |
| 12456 | 757 | 713 | Annexin IX isoform, *B. mori* | NP_00103684 | 7.8e-66 | Pfam00191 | Exocytosis (secretion); membrane fusion |
| 01210 | 3131 | 684 | Ubiquitin activating enzyme, *M. musculus* | AAF00149 | 1.4e-267 | Cl01490 | Protein degradation |
| 11934 | 137 | 678 | Neprilysin-like peptidase, *M. musculus* | AAG18446 | 4.0e-02 | Cl03208 | Metallopeptidase; membrane modification --**In spermatophore** |
| 12652 | 243 | 672 | Pyruvate kinase, *N. vitripennis* | XP_001600651 | 7.2 E-11 | Cl10032 | Regulate glycolysis |
| 11882 | 651 | 668 | Phosphatidylserine receptor, *S. purpuratus* | XP_001507689 | 2.1 E-28 | Cl10032 | Regulates glycolysis |
| 11938 | 825 | 663 | Adenylate kinase, *Ae. aegypti* | XP_001662844 | 1.0e-77 | Cl11962 | Nucleotide metabolism; regulates ATP – ADP phosphate transfer. |
| 10915 | 369 | 653 | Ribosomal protein L27 A, *I. scapulairs* | X AAY66881 | 9.8 e-57 | Pfam 0828 | Ribosomal translation mRNA to protein |
| 11582 | 697 | 652 | AGAP004777, *An. gambiae* | XP_001689085 | 2.4e-31 | [Pfam 6743](http://www.ncbi.nlm.nih.gov/Structure/cdd/cddsrv.cgi?ascbin=8&maxaln=10&seltype=2&uid=pfam06743&querygi=158297883&aln=1,439,0,71) | Serine/threonine kinase |
| 12620 | 292 | 641 | Poly A binding protein, *A. mellifera* | XP_396057 | 2.8e-25 | Cl02586 | Post-transcriptional gene expression |
| 12553 | 362 | 622 | 26 S Proteasome, *I. scapularis* | NP_001107478 | 1.0e-51 | COG5071 | Proteolysis: degrade unneeded/damaged proteins |
| 11854 | 622 | 590 | Zinc carboxypeptidase, *A. mellifera* | XP_001122133 | 1.9e-31 | Cd03860 | Metallopeptidase activity |
| 04814 | 489 | 580 | Calmodulin, *D. melanogaster* | NP_523710 | 1.3e-31 | Cd00051 | Calcium sensors; cell signaling |
| 12100 | 1327 | 575 | 17655p *D. melanogaster* | [AAN71083.1](http://www.ncbi.nlm.nih.gov/protein/25009824?report=genbank&log$=prottop&blast_rank=1&RID=RRMJWYNT016) | 1.9e -17 | None | Unknown |
| 10424 | 1575 | 574 | Kelch-domain protein *I. scapularis* | XM_00241549 | 8.0e-69 | Cd02701 | Kelch motif; protein – protein interactions |
| 11481 | 3381 | 573 | Myosin III *Ae. aegypti* | XP_001660540 | 1.9e-172 | Cd01379 | ATPases - muscle contraction |

^1^Excluding contigs with no matches in GenBank or e-values < 10 (unless matching proteins were also found in the MAG/TVD and/or spermatophore by LC MS/MS or evidence of significant change in expression).

^2^ Bp

^3^ Including match to species.

^4^ Found in the contig.

Species abbreviations: *Ae. aegypti*  = *Aedes aegypti; An. gambiae = Anopheles gambiae; A. mellifera* = *Apis mellifera; A. monolakensis = Argas monolakensis; B. mori* = *Bombyx mori; B. taurus = Bos taurus; D. variabilis = Dermacentor variabilis; D. melanogaster = Drosophila melanogaster; D. santomea = Drosophila santomea; G. gallus = Gallus gallus; H. sapiens = Homo sapiens; I. scapularis = Ixodes scapularis; L. major = Leishmania major; L. singoriensis = Lycosa singoriensis; M. musculus = Mus musculus; N. vitripennis = Nasonia vitripennis; O. parkeri = Ornithodoros parkeri; O. cuniculus = Oryctolagus cuniculus; R. norvegicus = Rattus norvegicus; R. solanacearum* = *Ralstonia solanacearum*;  *S. purpuratus = Strongylcentrotus purpuratus; T. infestans = Triatoma infestans; X. laevis= Xenopus laevis; X. tropicalis= Xenopus tropicalis*.
