# Supplementary material for: First Transcriptome of the Testis-Vas Deferens-Male Accessory Gland and Proteome of the Spermatophore from Dermacentor variabilis (Acari: Ixodidae)
Source: PLoS One. 2011 Sep 16;6(9):e24711. doi: 10.1371/journal.pone.0024711 (PMC3174968; doi:10.1371/journal.pone.0024711)
Supplement: Table S2 — Comparison of contigs from the male transcriptome identified by BLASTx with their corresponding proteins/peptides in the MAG/TVD and/or spermatophore identified by LC/MS/MS (excluding common house-keeping proteins).1,2 (DOCX) [file pone.0024711.s010.docx]

Table S2. Comparison of contigs from the male transcriptome identified by BLASTx with their corresponding proteins/peptides in the MAG/TVD and/or spermatophore identified by LC MS/MS (excluding common house-keeping proteins).­^1,2^

| **Category/Species** | **Contig No.** | | **E-value** | **Sig. P^3^** | **Accession No.** | **MW** | **MAG/TVD** | **SP** |
| --- | --- | --- | --- | --- | --- | --- | --- | --- |
| **Reproduction /reproductive** **processes (**GO: 0048609**)** | | | | | | | | |
| Sperm-associated antigen *C. familiaris* | | 06321 | 1.0 E-04 | No | XP537679 | 23 | Yes | No |
| Neprilysin sperm-associated metalloprotease *Ae. aegypti* | | 10643 | 1.0 E-04 | No | NP_649924 | 77 | No | Yes |
| Arylsulfatase (spermiogenesis protein) *Ae. aegypti* | | 00324 | 4.0 E-19 | No | XP_001648126 | 62 | Yes | Yes |
| Angiotensin converting enzyme (Dipeptidyl carboxypeptidase II), *I. scapularis* | | 12337 | 2.0 E-20 | No | EEC08311 | 66 | No | Yes |
| Guanine-nucleotide (GT) binding protein, *I. scapularis* | | 01502 | 3.7 E-46 | No | AAY66933 | 9 | Yes | No |
| Serine/threonine kinase-testis/spermiogenesis specific, *H. sapiens* | | 12696 | 4.8 E-03 | No | NP_443732 | 57 | Yes | No |
| Testis specific serine kinase, *S. purpuratus* | | 10811 | 3.0 E-06 | No | XP_787834 | 48 | Yes | No |
| ADAM disintegin-metalloproteinase putative, *I. scapularis* | | 00909 | 2.0 E-14 | No | XM_002416530 | 84 | Yes | No |
| Salivary secreted serine proteinase (Trypsin), *I. scapularis* | | 12380 | 2.6 E-36 | 1.00 | AAY66740 | 29 | Yes | Yes |
| Serine protease inhibitor (Serpin), *I. ricinus* | | 11029 | 1.2 E-91 | No | AAK61375 | 11 | No | Yes |
| Transmembrane protein – cell motility, *I. scapularis* | | 12307 | 0.323 | No | NP_521951 | 24 | Yes | No |
| Cyclophilin A *Rhipicephalus sanguineus* | | 03073 | 7.7 E-37 | No | ACX53895 | 18 | Yes | No |

| **Protein digestion (peptidases)** (**GO:0008233)** | | | | | | | |
| --- | --- | --- | --- | --- | --- | --- | --- |
| Anion trypsinogen, *E. caballus* | No | ------- | ----- | gi\|194209993 | 25 | Yes | Yes |
| Trypsin protease, *I. scapularis* | 12380 | 2.6 E-36 | 1.00 | AAY66740 | 29 | Yes | Yes |
| Secreted carboxypeptidase, *I. scapularis* | 02044 | 1.2E-08 | 0.99 | AAM93650 | 37 | No | Yes |
| Dipeptidyl carboxypeptidase II, *E. coli* | 10158 | 3.0 E-34 | 0.90 | XP_001376640 | 66 | Yes | No |
| Serine protease, *N. vitripennis* | 03075 | 6.3 E-12 | 1.00 | XP_001603816 | 39 | Yes | Yes |
| Putative serine protease, *Ae. aegypti* | 04459 | 3.9 E-07 | 1.00 | XP_313875 | 39 | No | Yes |
| Leucine aminopeptidase, *I. scapularis* | 12179 | 2.1 E-34 | No | XP002416112 | 20 | No | Yes |
| Cathepsin L-like cysteine proteinase B, *R. haemaphysaloides* | 05033 | 7.1 E-27 | 1.00 | AAQ16118 | 37 | Yes | No |
| Cathepsin L-like proteinase precursor, *R. microplus* | 02857 | 3.2 E-10 | 1.00 | AAQ16117 | 42 | Yes | No |
| Carboxypeptidase, *M. musculus* | 11854 | 3.0 E-36 | 0.97 | NP_808502 | 38 | No | Yes |

| **Protease inhibitors (GO: 0030414**) | | | | | | | |
| --- | --- | --- | --- | --- | --- | --- | --- |
| Intracellular cystatin, *D. variabilis* | 06091 | 9.4 E-04 | 1.00 | AAS55948 | 11 | Yes | Yes |
| Cystatin precursor, *H. longicornis* | 11343 | 9.6 E-06 | 1.00 | Q7M429 | 11 | No | Yes |
| Aprotinin analog, *B. taurus* | No | ---------- | No | gi\|360747 | 7 | No | Yes |
| Trypsin inhibitor, *E. caballus* | 01940 | 7.9 E-28 | No | XP_001495122 | 11 | No | Yes |

| **Hydrolases (GO: 0016787**) | | | | | | | |
| --- | --- | --- | --- | --- | --- | --- | --- |
| Hydrolase: mannosyl-3-phosphoglyc. phosphatase, *P. marinus* | No | --------- | No | gi\|190574514 | 29 | Yes | Yes |
| L-lactate dehydrogenase, *C. butyricum* | 00103 | 5.6 E-125 | 0.02 | NP_956167 | 31 | No | Yes |
| B-lactamase domain protein, *Clostridiales* spp. | No | -------- | ----- | gi\|217976598 | 23 | Yes | Yes |

| **Lipid digestion/lipases (GO: 0004806**) | | | | | | | |
| --- | --- | --- | --- | --- | --- | --- | --- |
| Phospholipase C gamma, *Ae. aegypti* | 06555 | 7.8 E-85 | No | XP_001649138 | 31 | Yes | Yes |
| Phospholipase D family, member 3, *H. sapiens* | 05091 | 6.8 E-12 | No | NP_001026866 | 57 | Yes | No |
| Phosphoinositide-specific phospholipase C, *N. rustica* | 09395 | 1.2 E-37 | No | CAA65127 | 57 | Yes | Yes |

| **Oxidative Stress** (**GO: 0055114)** | | | | | | | |
| --- | --- | --- | --- | --- | --- | --- | --- |
| Quinone oxireductase, *I. scapularis* | No | ------- | No | XP_002415000 | 42 | Yes | Yes |
| Glutamate dehydrogenase, *T. tengcongensis* | 00577 | 2.6 E-11 | No | NP_623754 | 59 | No | Yes |
| Thiamine pyrophosphate TTP-binding, ***A. thermophilum*** | No | -------- | No | ACM59987 | 26 | No | Yes |
| Putative thioredoxin, *D. variabilis* | 09407 | 4.7 E-10 | 0.99 | AAY66786 | 10 | Yes | Yes |
| Superoxide dismutase, *D. variabilis* | 03655 | 8.5 E-13 | No | AAY66847 | 16 | Yes | Yes |
| Glutathione S-transferase, *I. pacificus* | 07930 | 4.7 E-21 | No | AAT92159 | 22 | Yes | Yes |
| Phospholipid-hydroperoxide glutathione peroxidase, *R. microplus* | NO | ------- | No | ABA25916 | 19 | Yes | No |
| Thioredoxin peroxidase, *I. scapularis* | 11398 | 2.3 E-116 | 0.98 | AAY66580 | 10 | Yes | Yes |

| **Response to Environmental Stress** (**GO006950**) |  |  |  |  |  |  |  |
| --- | --- | --- | --- | --- | --- | --- | --- |
| Heat shock protein HSP-70, *L. menadoensis* | 11387 | 1.1 E-184 | No | ABS53145 | 50 | Yes | Yes |
| Heat shock protein HSP-70, *P. tristis* | 11192 | 4.8E-157 | No | ABQ12828 | 68 | Yes | Yes |
| Heat shock protein 90, *A. americanum* | 02357 | 5.5 E-61 | No | AAS45246 | 25 | Yes | Yes |
| Heat shock protein, *I. scapularis* | No | ------ | 1.00 | XM_00243361 | 68 | Yes | Yes |
| Heat shock protein, *P. yoelii* | No | --------- | 0.96 | XM_722180 | 74 | Yes | Yes |

| **Immune proteins** (**GO006955**) | | | | | | | |
| --- | --- | --- | --- | --- | --- | --- | --- |
| Lysozyme, chain A, *H. sapiens* | No | ------- | 1.00 | gi\|5821957 | 15 | Yes | Yes |
| Macrophage migration inhibitory factor, *D. variabilis* | No | ------- | No | ACF35547 | 11 | Yes | Yes |
| ML-domain protein, *B. mori* | 3157 | 1.6E-04 | 1.00 | NP_001040454 | 17 | No | Yes |
| Hemoglobin alpha, *O. cuniculus* | No | **-----** | ---- | NP_001075858 | 16 | Yes | Yes |
| Hemoglobin beta, *O. cuniculus* | No | **-----** | ----- | 660912A | 16 | Yes | Yes |

| **Cell Structure/Anatomical Structure/adhesion (GO0009653)** | | | | | | | |
| --- | --- | --- | --- | --- | --- | --- | --- |
| Actin 5 C, *D. melanogaster* | 12409 | 1.3 E-217 | No | AAP81255 | 42 | Yes | Yes |
| Alpha-tubulin, *D. melanogaster* | 11059 | 2.7 E-187 | No | XP_001606870 | 50 | Yes | Yes |
| Beta-tubulin, *D. melanogaster* | 08972 | 7.3 E-27 | No | NP_001036965 | 50 | Yes | Yes |
| Cytokeratin, 9 *H. sapiens* | 12389 | 2.1 E-03 | No | Q29426 | 62 | Yes | Yes |
| Keratin, *E. caballus* | 01860 | 3.7 E-32 | No | XP_001489884 | 58 | Yes | Yes |
| Calreticulin (cell adhesion-modulating protein), *D. variabilis* | 10500 | 1.8 E-176 | 1.00 | AAQ18697 | 47 | Yes | Yes |

^1^ Abbreviations: GB = Gene Bank; MAG/TVD = male accessory gland/testis vas deferens; MIF = Macrophage migration inhibitory factor; Mol. Wt. = molecular weight (kDa); Sig. P = Signal P; SP = spermatophore.

^2^ Species Abbreviations: Abbreviations as in Table 1. Additional abbreviations: *A. salmonicida* = *Aeromonas salmonicida; A. americanum = Amblyomma americanum; A. thermophilum* = *Anaerocellum thermophilum*; *C. butyricum =clostridium butyricum; C. fornicata* = *Crepidula fornicata*; *D. farinae = Dermatophagoides farinae; E. caballus = Equus caballus; E. coli = Escherichia coli; G. bryicola = Galianora bryicola;* *H. longicornis = Haemaphysalis longicornis; I. ricinus =* *Ixodes ricinus; L. menadoensis = Latimeria menadoensis; M. silvestris = Methylocella silvestris; P. ficiformis =Petrosia ficiformis; P. yoelii = Plasmodium yoelii;* *P. tristis* = *Plectreurys tristis; P. marinus = Prochlorococcus marinus; R. haemaphysaloides* =*Rhipicephalus haemaphysaloides; R. microplus = Rhipicephalus (Boophilus) microplus; R. leguminosarum = Rhizobium leguminosarum; T=tengcongensis =Thermoanaerobacter tengcongensis.*

^3^ www.cbs.dtu.dk/services/SignalP/
