# Supplementary material for: First Transcriptome of the Testis-Vas Deferens-Male Accessory Gland and Proteome of the Spermatophore from Dermacentor variabilis (Acari: Ixodidae)
Source: PLoS One. 2011 Sep 16;6(9):e24711. doi: 10.1371/journal.pone.0024711 (PMC3174968; doi:10.1371/journal.pone.0024711)
Supplement: Table S3 — Contigs in D. variabilis fed male accessory glands/testis/vas deferens associated with reproductive activity. (DOCX) [file pone.0024711.s011.docx]

Table S3. Contigs in *D. variabilis* fed male accessory glands/testis/vas deferens associated with reproductive activity.^1^

| **Contig No** | **E-value** | **Length** | **Sig. P**^2^ | **Best match nr database** | **Putative function** |
| --- | --- | --- | --- | --- | --- |
| 00588 | 5.1 E-42 | 388 | No | XP860742 | 26S protease sperm-associated protein SATA, *C. familiaris* |
| 00843 | 6.0 E-154 | 1868 | No | XM_001608159 | zinc metalloprotease *N. vitripennis*; *D. variabilis* GenBank FJ457900 |
| 01293 | 3.4 E-36 | 161 | No | AAD22773 | testis-enriched tyrosine phosphatase *M. musculus* |
| 01502 | 3.7 E-46 | 332 | No | AAY66933 | guanine-nucleotide-binding protein *I. scapularis* |
| 01950 | 4.0 E-72 | 402 | 0.99 | AAG45155 | 9.8 kDa basic protein (= ATP synthase) *A. hebraeum* |
| 02536 | 1.3 E-27 | 348 | 0.90 | XP001514254 | spermatogenesis associated, serine-rich 2, *X. laevis* |
| 03261 | 4.4 E-11 | 207 | No | ABK20019 | astacin-like metalloprotease a toxin precursor *Loxosceles intermedia* |
| 03882 | 3.7 E-32 | 384 | No | AAB94566 | ecdysone receptor, *A. americanum* |
| 06510 | 6.2 E-40 | 348 | No | XP00165847 | Gonadotropin inducible transcription factor, *Ae. aegypti* |
| 03048 | 5.0E-18 | 229 | 0.94 | XP_002734822 | prostaglandin F synthase, *E. caballus* |
| 07737 | 4.3 E-14 | 198 | 1.00 | NP_001016359 | insulin-like growth factor 2 mRNA binding protein, *G. gallus* |
| 08424 | 3.0 E-25 | 198 | No | ISCW017837 | G-protein coupled receptor, *I. scapularis* |
| 09571 | 1.5E-19 | 213 | No | AAC47275 | zinc metalloprotease ADAM (Kuzbanian), *D. melanogaster* |
| 10624 | 9.9 E-34 | 288 | 0.94 | AAG45156 | 10.4 kDa basic protein (= acylphosphatase), *A. hebraeum* |
| 11358 | 1.1 E-49 | 2495 | No | ABM92922 | male engorgement factor α, *D. variabilis* |
| 12359 | 6.3 E-15 | 446 | No | ABA62333 | subolesin ( = protective antigen 4D8), *D. marginatus* |

^_________________________________________________________________________________________________________________________________________________________^

^1^Abbreviations as in Tables S1 and S2. Additional abbreviations: *A. hebraeum* = *Amblyomma hebreum*; *C. familiaris* = *Canis familiaris; L. intermedia* = *Loxosceles intermedia.*

^2^www.cbs.dtu.dk/services/SignalP/
