# Supplementary material for: First Transcriptome of the Testis-Vas Deferens-Male Accessory Gland and Proteome of the Spermatophore from Dermacentor variabilis (Acari: Ixodidae)
Source: PLoS One. 2011 Sep 16;6(9):e24711. doi: 10.1371/journal.pone.0024711 (PMC3174968; doi:10.1371/journal.pone.0024711)
Supplement: Table S4 — Contigs in D. variabilis fed male accessory glands/testis/vas deferens associated with protein digestion by cysteine proteinases. (DOCX) [file pone.0024711.s012.docx]

Table S4. Contigs in *D. variabilis* fed male accessory glands/testis/vas deferens associated with protein digestion by cysteine proteinases.^1^

| **Contig No.** | **E-value** | **Length** | **Sig. P^2^** | **Best match nr database** | **Putative function** |
| --- | --- | --- | --- | --- | --- |
| 00636 | 4.0 E-37 | 425 | 0.98 | EEC05117 | calcium-dependent cysteine protease, *I. scapularis* |
| 00637 | 1.0 E-41 | 356 | No | EEC05117 | calcium-dependent cysteine protease, *I. scapularis* |
| 00689 | 4.5 E-19 | 197 | 0.96 | ABO26563 | cathepsin B-like cysteine protease form 1, *I. ricinus* |
| 01578 | 2.3 E-18 | 508 | 0.84 | 001120458 | calpain-C calcium-activated neutral proteinase, *A. mellifera* |
| 09416 | 1.8 E-22 | 241 | 0.99 | AAU81589 | cysteine protease, *Petunia* x hybrid |
| 09488 | 1.2 E-16 | 223 | 0.93 | XP_001238322 | calcium-dependent cysteine proteinase, *An. gambiae* |

^1^Abbreviations as in Tables S1 and S2. Additional abbreviations: *I. ricinus* = *Ixodes ricinus.*

^2^www.cbs.dtu.dk/services/SignalP
