# Supplementary material for: First Transcriptome of the Testis-Vas Deferens-Male Accessory Gland and Proteome of the Spermatophore from Dermacentor variabilis (Acari: Ixodidae)
Source: PLoS One. 2011 Sep 16;6(9):e24711. doi: 10.1371/journal.pone.0024711 (PMC3174968; doi:10.1371/journal.pone.0024711)
Supplement: Table S5 — Contigs in D. variabilis fed male accessory glands/testis/vas deferens associated with protein digestion by serine proteinases/carboxypeptidases. (DOCX) [file pone.0024711.s013.docx]

Table S5. Contigs in *D. variabilis* fed male accessory glands/testis/vas deferens associated with protein digestion by serine proteinases/carboxypeptidases^1^.

| **Contig No** | **E-value** | **Length** | **Sig. P^2^** | **Best match nr database** | **Putative function** |
| --- | --- | --- | --- | --- | --- |
| 00414 | 3.0 E-10 | 298 | 1.00 | EEC00959 | serine proteinase, *I. scapularis*. |
| 01909 | 5.9 E-08 | 426 | 0.95 | XP_001652075 | clip-domain serine protease subfamily B, *Ae. aegypti* |
| 02501 | 1.7 E-07 | 444 | 0.99 | AAl79565 | midgut serine protease-1, *R. appendiculatus* |
| 04099 | 7.9 E-10 | 752 | No | EDL89997 | serine proteinase precursor, *R. norvegicus* |
| 06754 | 1.4 E-09 | 224 | 1.00 | NP_001037368 | serine proteinase-like protein, *B. mori* |
| 07209 | 8.2 E-10 | 229 | 0.98 | AAZ41365 | clip-domain trypsin-like serine peptidase 1, *L. salmonis* |
| 08489 | 1.0 E-16 | 182 | 0.99 | EEC14392 | serine carboxypeptidase, *I. scapularis* |
| 10844 | 2.0 E-81 | 1360 | 0.89 | EDP28804 | zinc carboxypeptidase, *B. malayi* |
| 11854 | 3.0 E-36 | 622 | 0.99 | NP_808502 | carboxypeptidase, *M. musculus* |
| 12304 | 1.2 E-08 | 216 | 0.99 | AAM93650 | secreted serine carboxypeptidase, *I. scapularis* |
| 12329 | 6.2 E-18 | 886 | 1.00 | AAL15154 | serine proteinase, *C. dilutus* |
| 12381 | 2.0 E-17 | 283 | 0.98 | EEC04241 | serine proteinase, putative, *I. scapularis* |

^1^Abbreviations as in Tables S1 and S2. Additional abbreviations: *C. dilutus* = *Creontiadis dilutes; L. salmonis = Lepeophtheirus salmonis.*

^2^www.cbs.dtu.dk/services/SignalP
