# Supplementary material for: First Transcriptome of the Testis-Vas Deferens-Male Accessory Gland and Proteome of the Spermatophore from Dermacentor variabilis (Acari: Ixodidae)
Source: PLoS One. 2011 Sep 16;6(9):e24711. doi: 10.1371/journal.pone.0024711 (PMC3174968; doi:10.1371/journal.pone.0024711)
Supplement: Table S6 — Contigs in D. variabilis fed male accessory glands/testis/vas deferens associated with control of reproductive activity by proteinase inhibitors. (DOCX) [file pone.0024711.s014.docx]

Table S6. Contigs in *D. variabilis* fed male accessory glands/testis/vas deferens associated with control of reproductive activity by proteinase inhibitors^1^.

| **Contig No** | **E-value** | **Length** | **Sig. P** | **Best match nr database** | **Putative function** |
| --- | --- | --- | --- | --- | --- |
| 01313 | 2.0 E-21 | 546 | No | EEC14374 | serpin2 precursor, putative, *I. scapularis* |
| 03201 | 5.5 E-24 | 864 | 1.00 | ABC25074 | serine protease inhibitor 4, *G. morsitans morsitans* |
| 03396 | 1.6E-13 | 239 | 1.00 | AAN10067 | Kunitz-type serine proteinase inhibitor, *A. caninum* |
| 03569 | 1.2 E-24 | 222 | 1.00 | AAK61377 | serine proteinase inhibitor serpin-3, *R. appendiculatus* |
| 06151 | 1.2 E-08 | 220 | 1.00 | [AY312432](http://www.ncbi.nlm.nih.gov/nuccore/32351459?ordinalpos=1&itool=EntrezSystem2.PEntrez.Sequence.Sequence_ResultsPanel.Sequence_RVDocSum) | serine proteinase inhibitor serpin-1, *R. microplus* |
| 09308 | 7.0 E-12 | 200 | No | EEC19557 | serine proteinase inhibitor serpin-4 precursor, *I. scapularis* |
| 10596 | 2.0 E-09 | 961 | ----- | EDM76065 | serine protease inhibitor family, *P. pacifica* |
| 10958 | 9.9 E-08 | 441 | No | CAB55818 | serine proteinase inhibitor serpin-1, *I. ricinus* |
| 11029 | 1.2 E-91 | 1311 | No | CAB55818 | serine protease inhibitor*, I. ricinus* |
| 11032 | 5.9 E-11 | 918 | 0.98 | ABS87361 | lospin 8 (= serpin), *A. americanum* |

^1^Abbreviations as in Tables S1 and S2. Additional abbreviations: *A. caninum* = *Ancylostoma caninum*; *G. morsitans* = *Glossina morsitans*; *P. pacifica = Pleistocystis pacifica.*
