# Supplementary material for: First Transcriptome of the Testis-Vas Deferens-Male Accessory Gland and Proteome of the Spermatophore from Dermacentor variabilis (Acari: Ixodidae)
Source: PLoS One. 2011 Sep 16;6(9):e24711. doi: 10.1371/journal.pone.0024711 (PMC3174968; doi:10.1371/journal.pone.0024711)
Supplement: Table S7 — Contigs in D. variabilis fed male accessory glands/testis/vas deferens associated with control of carbohydrate digestion by amylases/hydrolases. (DOCX) [file pone.0024711.s015.docx]

Table S7. Contigs in *D. variabilis* fed male accessory glands/testis/vas deferens associated with control of carbohydrate digestion by amylases/hydrolases^1^.

| **Contig No** | **E-value** | **Length** | **Sig. P**^2^ | **Best match nr database** | **Putative function** |
| --- | --- | --- | --- | --- | --- |
| 00103 | 5.6 E-125 | 1492 | 0.95 | NP_956167 | L-lactate dehydrogenase, *C. butyricum* |
| 07399 | 7.8 E-16 | 212 | No | XP_001070897 | PREDICTED: Maltase-glucoamylase, intestinal, *R. norvegicus* |
| 08302 | 5.8 E -15 | 730 | No | AAI15035 | maltase-glucoamylase, partial, *M. mulatta* |

^1^Abbreviations as in Tables S1 and S2. Additional abbreviations:*.* *C. butyricum* = *Clostridium butyricum*; *M. mulatta = Macaca mulatta.*

^2^www.cbs.dtu.dk/services/SignalP/
