# Supplementary material for: First Transcriptome of the Testis-Vas Deferens-Male Accessory Gland and Proteome of the Spermatophore from Dermacentor variabilis (Acari: Ixodidae)
Source: PLoS One. 2011 Sep 16;6(9):e24711. doi: 10.1371/journal.pone.0024711 (PMC3174968; doi:10.1371/journal.pone.0024711)
Supplement: Table S8 — Contigs in D. variabilis fed male accessory glands/testis/vas deferens associated with control of lipid digestion/lipid storage by lipases. (DOCX) [file pone.0024711.s016.docx]

Table S8. Contigs in *D. variabilis* fed male accessory glands/testis/vas deferens associated with control of lipid digestion/lipid storage by lipases^1^.

| **Contig No** | **E-value** | **Length** | **Sig. P**^2^ | **Best match nr database** | **Putative function** |
| --- | --- | --- | --- | --- | --- |
| 00160 | 9.8 E-30 | 397 | No | XP_968978 | intracellular membrane Ca^++^ independent phospholipase A2γ,  *T. castaneum* |
| 00806 | 9.4 E-14 | 217 | 1.00 | EDP302801 | carboxyl ester lipase, *X. laevis* |
| 02434 | 2.3 E 18 | 550 | No | CAK50252 | phospholipase B-like protein, *D. discoideum* |
| 02461 | 3.0E-11 | 215 | 0.88 | AAV842 | triacylglycerol acid lipase, *C. sonorensis* |
| 04115 | 1.0 E-10 | 399 | No | ABI52780 | lysophospholipase, *A. monolakensis* |
| 05091 | 6.8 E-12 | 216 | No | NP_001026866 | phospholipase D family, member 3, *H. sapiens* |
| 06555 | 7.8 E-85 | 1276 | No | XP_001649993 | lipase 1 precursor, *Ae. aegypti* |
| 09395 | 1.2 E-37 | 234 | No | CAA65127 | phosphoinositide-specific phospholipase C, *N. rustica* |
| 03073 | 5.0 E-36 | 241 | No | AAP35065 | der f mal f 6 allergen lipid binding, *D. farinae* |
| 8455 | 2.0 E-12 | 198 | No | AAD42075 | phospholipase A-2-activating protein, *H. sapiens* |

^1^Abbreviations as in Tables S1 and S2. Additional abbreviations: *C. sonorensis* = *Culicoides sonorensis; D. discodeum = Dictyostelium discodeum; N. rustica* = *Nicotiana rustica*; *T. castaneum* = *Tribolium castaneum.*

^2^www.cbs.dtu.dk/services/SignalP/
