# Supplementary material for: First Transcriptome of the Testis-Vas Deferens-Male Accessory Gland and Proteome of the Spermatophore from Dermacentor variabilis (Acari: Ixodidae)
Source: PLoS One. 2011 Sep 16;6(9):e24711. doi: 10.1371/journal.pone.0024711 (PMC3174968; doi:10.1371/journal.pone.0024711)
Supplement: Table S9 — Contigs in D. variabilis fed male accessory glands/testis/vas deferens associated with oxidative stress. (DOCX) [file pone.0024711.s017.docx]

Table S9. Contigs in *D. variabilis* fed male accessory glands/testis/vas deferens associated with oxidative stress^1^.

| **Contig No** | **E-value** | **Length** | **Sig. P**^2^ | **Best match nr database** | **Putative function** |
| --- | --- | --- | --- | --- | --- |
| 00433 | 5.8 E-19 | 547 | No | NP_001040246 | glutaredoxin, *B. mori* |
| 00475 | 1.8 E-29 | 353 | No | NP_001005950 | glutaredoxin, *D. rerio* |
| 00577 | 2.6 E-11 | 780 | No | NP_623754 | glutamate dehydrogenase, *T. tengcongensis* |
| 04094 | 6.2 E-20 | 1212 | No | NP_037502 | oxidative stress induced growth inhibitor, *H. sapiens* |
| 05014 | 2.0 E-16 | 227 | No | XP_793267 | predicted: microsomal glutathione-S-transferase, *S. purpuratus* |
| 05072 | 5.5 E-09 | 199 | No | AAT92159 | glutathione-S-transferase 2, *I. pacificus* |
| 09214 | 4.5 E-12 | 102 | No | AAD15991 | glutathione S-transferase, *R. microplus* |
| 11859 | 3.2 E-30 | 888 | 1.00 | XP_419079 | thioredoxin domain containing 4 (endoplasmic reticulum), *G. gallus* |
| 11946 | 1.8 E-21 | 342 | No | NP_787019 | glutathione-S-transferase, *B. taurus* |

^1^Abbreviations as in Tables 3. Additional abbreviations: *D. rerio* = *Danio rerio; I. pacificus* = *Ixodes pacificus.*

^2^www.cbs.dtu.dk/services/SignalP/
