# Supplementary material for: First Transcriptome of the Testis-Vas Deferens-Male Accessory Gland and Proteome of the Spermatophore from Dermacentor variabilis (Acari: Ixodidae)
Source: PLoS One. 2011 Sep 16;6(9):e24711. doi: 10.1371/journal.pone.0024711 (PMC3174968; doi:10.1371/journal.pone.0024711)
Supplement: Table S10 — Contigs in D. variabilis fed male accessory glands/testis/vas deferens associated with environmental stress. (DOCX) [file pone.0024711.s018.docx]

Table S10. Contigs in *D. variabilis* fed male accessory glands/testis/vas deferens associated with environmental stress^1^.

| **Contig No** | **E-value** | **Length** | **Sig. P**^2^ | **Best match nr database** | **Putative function** |
| --- | --- | --- | --- | --- | --- |
| 00189 | 1.5 E-26 | 294 | No | NP_006810 | stress-induced-phosphoprotein 1 Hsp70/90-org- protein, *H. sapiens* |
| 06257 | 1.8 E-62 | 450 | No | XP_00165238 | methylmalonate semialdehyde dehydrogenase, *Ae. aegypti* |
| 12677 | 5.6e-139 | 1583 | No | NP_006588 | heat shock protein, *O. anatinus* |
| 12744 | 4.8E-73 | 0855 | No | NP_989360 | stress-induced-phosphoprotein Hsp70/90-organizing protein, *X. tropicalis* |

^1^Abbreviations as in Tables S1 and S2. Additional abbreviations: *O. anatinus* = *Ornithorhynchus anatinus*

^2^www.cbs.dtu.dk/services/SignalP/
