# Supplementary material for: First Transcriptome of the Testis-Vas Deferens-Male Accessory Gland and Proteome of the Spermatophore from Dermacentor variabilis (Acari: Ixodidae)
Source: PLoS One. 2011 Sep 16;6(9):e24711. doi: 10.1371/journal.pone.0024711 (PMC3174968; doi:10.1371/journal.pone.0024711)
Supplement: Table S11 — Contigs in D. variabilis fed male accessory glands/testis/vas deferens associated with innate immunity. (DOCX) [file pone.0024711.s019.docx]

Table S11. Contigs in *D. variabilis* fed male accessory glands/testis/vas deferens associated with innate immunity^1^.

| **Contig No** | **E-value** | **Length** | **Sig. P**^2^ | **Best match nr database** | **Putative function** |
| --- | --- | --- | --- | --- | --- |
| 00983 | 5.0E-27 | 551 | 1.00 | AAM88421 | OMFREP (Fibrinogen related domain protein), *O. moubata* |
| 04057 | 1.2E-19 | 220 | 0.99 | AAQ93650 | ixoderin (=lectin) precursor, *I. ricinus* |
| 05417 | 1.8E-12 | 225 | 1.00 | ABW08118 | defensin (varisin), *D. variabilis* |
| 07749 | 6.2 E-16 | 177 | 0.95 | XP_001121830 | basigin matrix metalloprotease (Immune superfamily), *A. mellifera* |
| 07964 | 5.0E-29 | 193 | 0.86 | XP_001654670 | lectin, *Ae. aegypti* |
| 12360 | 1.8E-31 | 372 | 1.00 | AAP93589 | lectin: dorin M precursor, *O. moubata* |
| 12361 | 2.2E-37 | 372 | 0.99 | AAQ93650 | ixoderin (=lectin) precursor, *I. ricinus* |

^1^Abbreviations as in Tables S1 and S2

^2^www.cbs.dtu.dk/services/SignalP/
