# Supplementary material for: First Transcriptome of the Testis-Vas Deferens-Male Accessory Gland and Proteome of the Spermatophore from Dermacentor variabilis (Acari: Ixodidae)
Source: PLoS One. 2011 Sep 16;6(9):e24711. doi: 10.1371/journal.pone.0024711 (PMC3174968; doi:10.1371/journal.pone.0024711)
Supplement: Table S12 — Contigs in D. variabilis fed male accessory glands/testis/vas deferens associated with cytoskeletal/cell adhesion. (DOCX) [file pone.0024711.s020.docx]

Table S12. Contigs in *D. variabilis* fed male accessory glands/testis/vas deferens associated with cytoskeletal/cell adhesion^1^.

| **Contig No** | **E-value** | **Length** | **Sig. P^1^** | **Best match nr database** | **Putative function** |
| --- | --- | --- | --- | --- | --- |
| 01679 | 7.5 E-51 | 778 | No | AAM64112 | gelsolin-like allergen Der actin-binding protein, *D. farinae* |
| 05344 | 4.0 E-10 | 194 | 0.56 | ACF35545 | putative cement protein, *D. variabilis* |
| 05925 | 1.7 E-23 | 224 | No | AAA58407 | cadherin-associated protein, vinculin family, *H. sapiens* |
| 06178 | 2.9 E-15 | 232 | No | XP_001660147 | cadherin, *Ae. aegypti* |
| 06925 | 1.3 E-13 | 208 | 1.00 | XP_311354 | integrin, *An. gambiae* |
| 07300 | 5.7 E-14 | 195 | No | XP_001601620 | fascilin-1 precursor, *N. vitripennis* |
| 10467 | 1.8 E-55 | 1668 | No | AAL75584 | tetraspanin-like protein, *D. variabilis* |
| 11051 | 2.7 E-187 | 1140 | No | XP_001606870 | alpha tubulin, *N. vitripennis* |
| 11668 | 4.5 E-123 | 2015 | No | NP_112729 | alpha 3 type IV collagen binding protein isoform 2, *H. sapiens* |
| 12680 | 2.8E-67 | 702 | No | XP_001177924 | similar to 34/67 kD laminin binding protein, *S. purpuratus* |

^1^Abbreviations as in Tables S1 and S2

*.*

^2^www.cbs.dtu.dk/services/SignalP/
